# Interferon-alpha treatment rapidly clears Hepatitis E virus infection in humanized mice

**DOI:** 10.1038/s41598-017-07434-y

**Published:** 2017-08-15

**Authors:** Martijn D. B. van de Garde, Suzan D. Pas, Gertine W. van Oord, Lucio Gama, Youkyung Choi, Robert A. de Man, Andre Boonstra, Thomas Vanwolleghem

**Affiliations:** 1 Department of Gastroenterology and Hepatology, Erasmus University Medical Center, Rotterdam, The Netherlands; 2 Department of Viroscience, Erasmus University Medical Center, Rotterdam, The Netherlands; 3 Laboratory of Experimental Medicine and Pediatrics, Faculty of Medicine and Health Sciences, University of Antwerp and Department of Gastroenterology and Hepatology, Antwerp University Hospital, Antwerp, Belgium; 4 Department of Molecular and Comparative Pathobiology, The Johns Hopkins University School of Medicine, Baltimore, Maryland USA; 5 Division of Viral Hepatitis, The Center for Disease Control and Prevention, Atlanta, Georgia USA

## Abstract

Antiviral treatment options for chronic Hepatitis E Virus (HEV) infections are limited and immunological determinants of viral persistence remain largely unexplored. We studied the antiviral potency of pegylated interferon-α (pegIFNα) against HEV infections in humanized mice and modelled intrahepatic interferon stimulated gene (ISG) responses. Human gene expression levels in humanized mouse livers were analyzed by qPCR and Nanostring. Human CXCL10 was measured in mouse serum. HEV genotype 3 (gt3) infections were cleared from liver and feces within 8 pegIFNα doses in all mice and relapsed after a single pegIFNα injection in only half of treated animals. Rapid viral clearance by pegIFNα was confirmed in HEV gt1, but not in Hepatitis B Virus infected animals. No ISG induction was observed in untreated HEV gt3 and gt1 infected humanized livers compared to control chimeric mice, irrespective of the human hepatocyte donor, viral isolate or HEV infection duration. Human specific ISG transcript levels in mouse liver increased significantly after pegIFNα treatment and induced high circulating human CXCL10 in mouse serum. In conclusion, HEV gt1 and gt3 infections do not elicit innate intrahepatic immune responses and remain highly sensitive to pegIFNα in immunocompromised humanized mice.

## Introduction

Hepatitis E Virus (HEV) infections are emerging in western countries^1^. HEV is a non-enveloped positive-sense single-stranded RNA virus, belonging to the family *Hepeviridae* within the genus *Orthohepevirus* ^2^. Transmission mainly occurs through the fecal-oral route via contaminated water in developing countries or through the consumption of undercooked meat in industrialized countries^3^. Seven different genotypes have been described so far, of which genotype (gt) 1 and 3 are most prevalent in humans^2^. In healthy individuals, HEV mostly resolves spontaneously without severe symptoms, but pregnant women seem to be at risk of developing fulminant liver failure by HEV gt1 with mortality rates up to 25%^4, 5^. On the other hand, increasing rates of chronic gt3 infections have been described in immunocompromised patients in Europe, resulting in progressive liver fibrosis and cirrhosis^6–8^. These data indicate that host pathogen interactions differ between both genotypes.

Antiviral treatment options for chronic HEV infected immunocompromised patients are limited. Ribavirin (RBV) leads to sustained viral responses in roughly 75% of patients, but is hampered by RBV-induced anemia and the need for recombinant erythropoietin injections or transfusions in more than half of patients^9, 10^. As an alternative, pegylated interferon-alpha (pegIFNα) has been administered to a few patients in doses comparable to Hepatitis C virus (HCV) treatment regimens^10, 11^. However, factors associated with interferon (IFN)-susceptibility, the optimal pegIFNα dose or treatment duration have not been investigated *in vivo*.

The anti-HEV effects of IFNα *in vitro* differ according to the target cell and viral strain used. *In vitro* HEV models consist of human hepatoma and lung adenocarcinoma cell-lines, in which replication of subgenomic or full length replicons and seldom intact patient-derived viruses are studied^12–16^. Patient-derived HEV gt3 cultures show slow viral propagation, whereas HEV gt1 can only be cultured *in vitro* after induction of endoplasmic reticulum stress in the host cell line^15–17^. While HEV gt1 replication has been shown to be adequately suppressed by exogenous IFNα, HEV gt3 replication has not^18–20^. In addition, viral inhibition of the interferon stimulated gene (ISG) responses have been described as a determining factor for IFNα susceptibility *in vitro* ^21^. As the studied host cells are either no target cells *in vivo* (A549 cells) or are hampered by defects in their innate immune signaling (Huh7 and Huh7.5), the host response towards genuine patient-derived HEV in differentiated human hepatocytes remains to be established^18, 22^. In addition, several clinical observations are not matched by *in vitro* viral replication data. HEV containing an *in vivo* RBV acquired mutation (K1383N), showed conflicting results *in vitro* with decreased viral replication and increased RBV-sensitivity^23^. Furthermore, the antiviral efficacy of sofosbuvir against HEV showed discrepancies in different *in vitro* and *in vivo* studies^24–27^.

Recently, we and others have shown that human-liver chimeric mice can be used to study HEV infection in differentiated human hepatocytes *in vivo* ^15, 28, 29^. Here, we examined baseline ISG expression levels and susceptibility to pegIFNα in HEV gt1 and gt3 infected humanized mice. We demonstrate that HEV gt1 infections lead to higher virus loads in mouse feces, bile and liver compared to HEV gt3 infections, without the induction of intrahepatic human innate immune responses. Both HEV genotypes, but not Hepatitis B virus (HBV), are cleared after a few doses of pegIFNα *in vivo*, an effect accompanied by a clear increase of human ISG transcript levels in liver and of circulating human CXCL10 levels in mouse serum.

## Results

### Higher viral burden in HEV gt1 compared to gt3 infected human-liver chimeric mice

**Figure 1:**
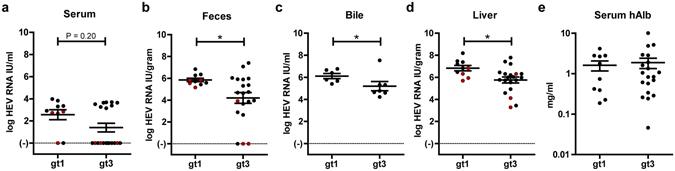
Higher viral loads in HEV gt1 compared to HEV gt3 infected mice despite similar degrees of human chimerism. Comparison of peak HEV RNA levels as measured by qRT-PCR in serum (**a**) and in feces (**b**). At sacrifice viral titers of HEV gt3 and gt1 infected mice were compared in bile (**c**, n = 7 and n = 6, respectively) and in liver (**d**). Human albumin levels were determined in mouse serum to quantify degree of chimerism at euthanasia (**e**). *P < 0.05, n = 20 for HEV gt3 and n = 10 for HEV gt1 (**a**,**b**,**d**,**e**). Data are pooled from 2, 6, and 14 weeks infection experiments. Red dots indicate mice who received a diluted HEV inoculum (**a**–**d**).

Humanized UPA^+/+^NOG mice were i.v. inoculated with a filtered feces suspension containing either HEV gt3 or HEV gt1 and were observed for 2, 6 or 14 weeks until euthanization. Infected mice were housed individually to prevent inter-mice contamination. During the infection course a higher percentage of HEV gt1 infected mice presented viremia, but the peak viral load in serum was similar to HEV gt3 infected mice (2.6 ± 0.4 and 1.4 ± 0.4 log HEV RNA IU/ml, respectively, Fig. 1a). The peak HEV RNA load in feces was significantly higher in HEV gt1, compared to HEV gt3 infected mice (5.9 ± 0.2 and 4.2 ± 0.5 log HEV RNA IU/gram, respectively, P = 0.029; Fig. 1b). HEV gt1 infected mice also had higher viral loads in bile (6.1 ± 0.2 vs. 5.2 ± 0.4 log HEV RNA IU/ml, respectively, P = 0.038; Fig. 1c) and liver (6.8 ± 0.2 vs 5.8 ± 0.3 log HEV RNA IU/gram, respectively, P = 0.015; Fig. 1d) at euthanasia, despite similar levels of serum human albumin compared to HEV gt3 infected mice, indicative for similar degrees of human chimerism (1.6 ± 0.4 and 1.9 ± 0.5 mg/ml, respectively, Fig. 1e). Despite lower absolute HEV gt1 inocula compared to HEV gt3, animals challenged with undiluted feces suspensions demonstrated similar results reaching higher HEV gt1 RNA levels in bile (P = 0.038), liver (P = 0.006), and feces (P = 0.06) compared to HEV gt3 RNA levels. These results point to a higher *in vivo* virulence of HEV genotype 1 compared genotype 3.

### No induction of intrahepatic innate immune responses in HEV gt1 or gt3 infected human-liver chimeric mice

**Figure 2:**
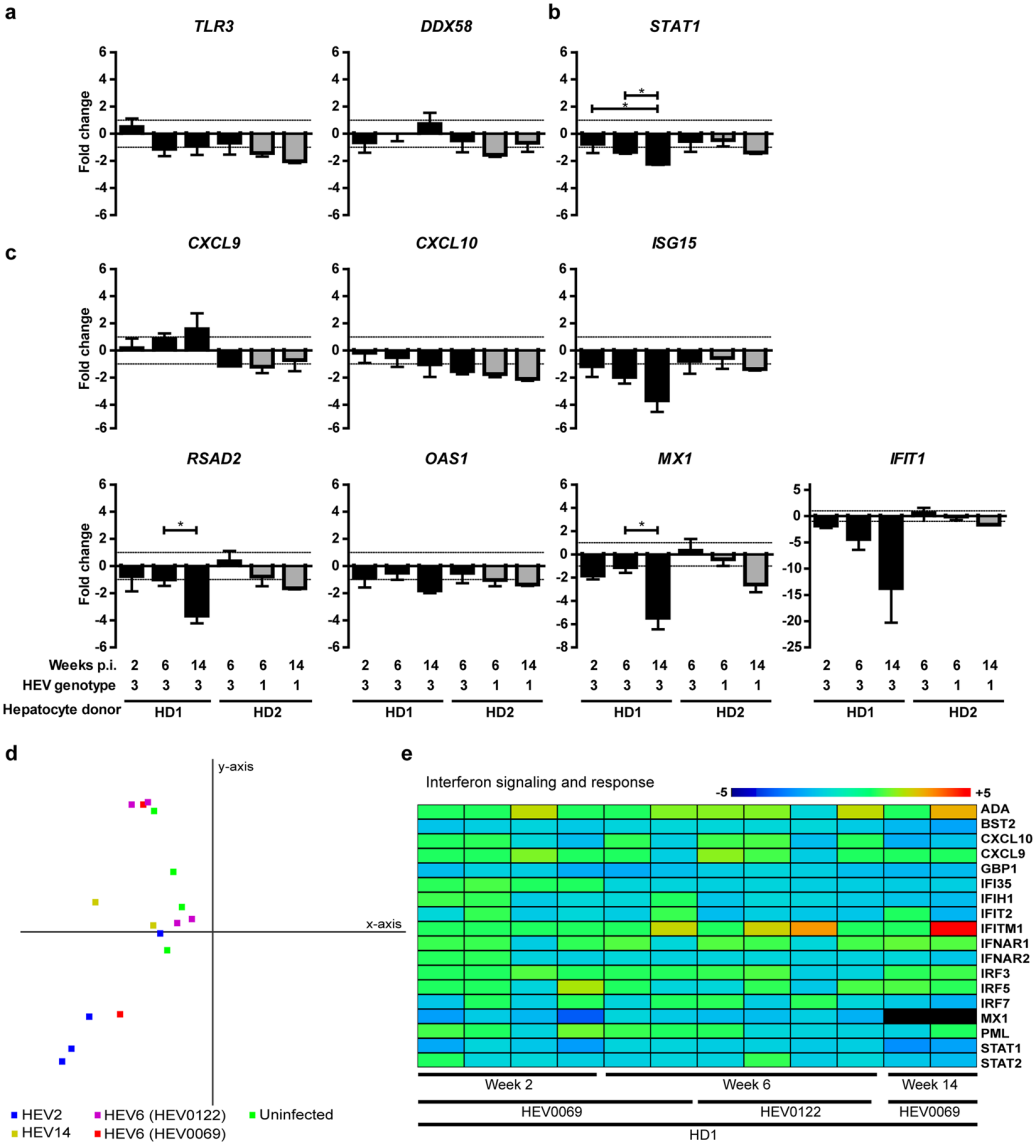
Minimal intrahepatic interferon-stimulated gene induction in HEV infected human-liver chimeric mice, between weeks 2 to 14 post infection. Whole chimeric-liver RNA was isolated from HEV gt3 and gt1 infected mice and analyzed for the human specific gene expression of sensing molecules *TLR3* and *DDX58* (**a**), transcription factor *STAT1* (**b**), and interferon stimulated genes *CXCL9*, *CXCL10*, *ISG15*, *RSAD2*, *OAS1*, *MX1* and *IFIT1* (**c**) using qRT-PCR. Groups consist of n = 4, 6, 4, 3, 6 and 4 mice from left to right (**a**–**c**). Given values on y-axes are fold changes over HEV RNA negative chimeric-livers transplanted with the same hepatocyte donor. X-axes shows weeks post infection, HEV genotype, and hepatocyte donor. Significance was assessed within groups of the same hepatocyte donor using Krukskal-Wallis one-way Anova with Dunnett’s Multiple comparison test. *P < 0.05, Gray bars indicate HEV gt1, black bars HEV gt3 (**a**–**c**). In-depth human gene expression analysis was performed on RNA from chimeric mouse livers before infection, and after 2, 6 and 14 weeks of HEV gt3 infection using nCounter® Human Immunology V2 panel. Principal component 1 (x-axis) and 2 (y-axis) comprise 49% of the variance between samples using all non-cross reactive genes (**d**). Uninfected samples are indicated in green, infected samples are indicated in blue, red (HEV0069), purple (HEV0122) and yellow and by the number of weeks infected HEV2, HEV6, HEV6, HEV14, respectively (**d**). Heatmap shows fold change over average of 4 uninfected mice for interferon signaling and response genes (**e**). Gene legend is indicated on the right side and sample legend below the heatmap (**e**). Dark red indicates ≥ 5 fold change, and dark blue ≤ 5 fold change (**e**).

Because of the HEV gt1 and gt3 clinical differences^4, 6–8^ and different viral burdens in humanized mice, we examined the human host response in chimeric livers 2, 6, or 14 weeks after infection with either HEV gt1 or gt3. Using qRT-PCR we could not detect a significant increase in transcript levels of alpha or beta IFNs (data not shown), pathogen recognition receptors *TLR3* and *DDX58* (Fig. 2a), transcription factor *STAT1* (Fig. 2b), or ISGs *CXCL9*, *CXCL10*, *ISG15*, *RSAD2*, *OAS1*, *MX1*, and *IFIT1* (Fig. 2c). Furthermore, longer duration of HEV gt3, but not HEV gt1 infection led to significantly decreased *STAT1*, *RSAD2* and *MX1* expression levels in the liver (Fig. 2b+c). None of these human transcripts were detected in non-chimeric mouse livers.

In order to evaluate a broader number of genes, Nanostring analysis of 594 human specific immunology-related genes was performed on chimeric (serum hAlb 2.5 ± 0.8 mg/ml) gt3 HEV-infected livers (6.1 ± 0.25 log HEV RNA IU/gr) at different time points post infection. Human transcript specificity was confirmed by including RNA from 3 non-chimeric livers and led to the removal of 50 cross-reactive genes from further analyses. Based on set criteria (<100 relative RNA counts and below four times the standard deviation in all samples), 255 genes were defined as non-expressed. Principal component analyses did not reveal clustering of samples (Fig. 2d). Of 18 genes related to interferon signaling and response, none showed consistent upregulation compared to non-infected chimeric mice (Fig. 2e). Down regulation of *STAT1* and *MX1* as observed by qRT-PCR, was confirmed in the Nanostring gene expression data (Fig. 2b+c,e). Taken together, these data show that ongoing HEV gt1 or gt3 replication for up to 14 weeks does not elicit an innate immune response in human hepatocytes *in vivo*.

### HEV but not HBV is sensitive to pegIFNα-2a treatment in human-liver chimeric mice

**Figure 3:**
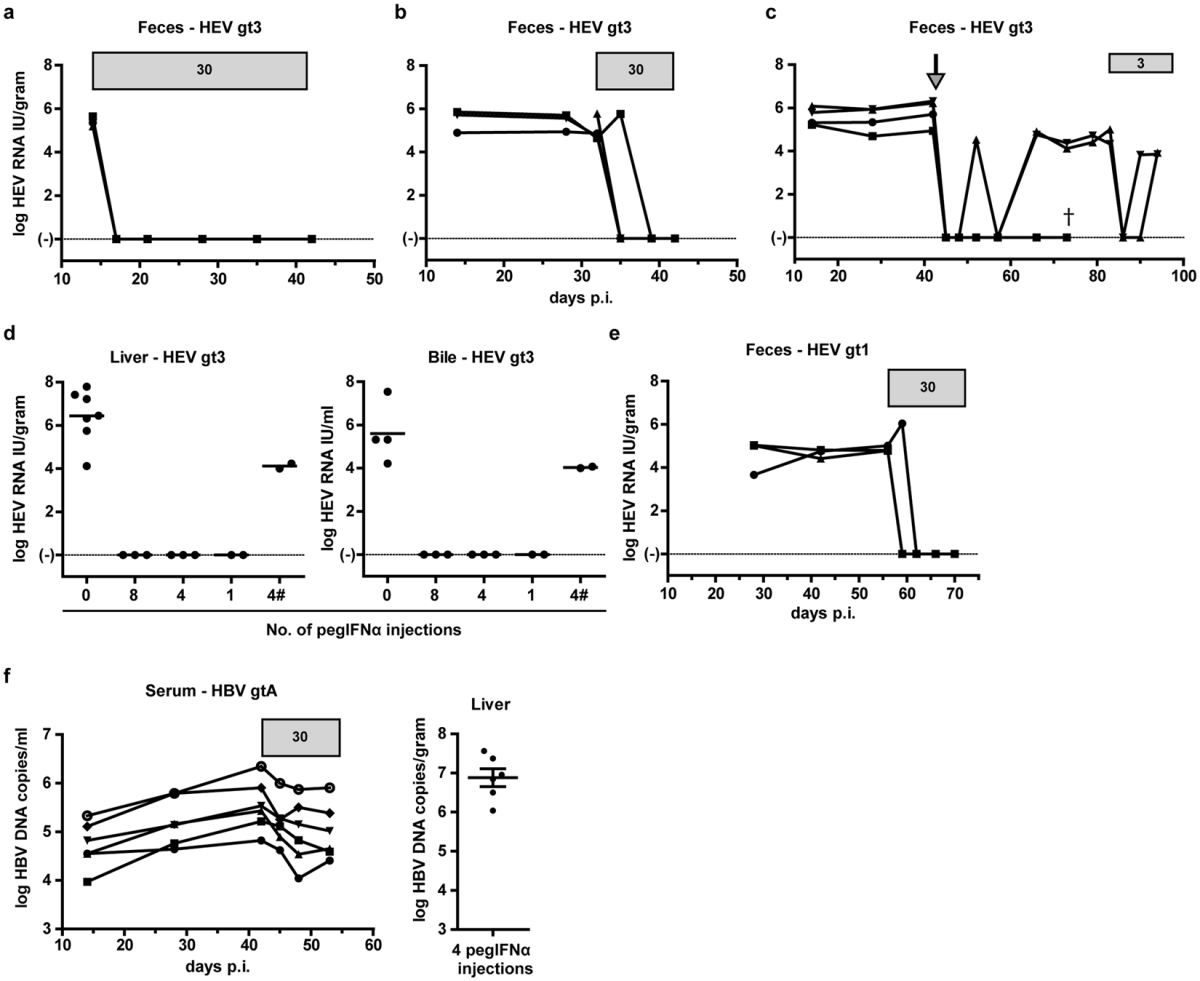
HEV is more sensitive than HBV to pegIFNα treatment in human-liver chimeric mice. HEV RNA was measured by qRT-PCR in feces of human-liver chimeric mice infected with HEV gt3 or HEV gt1 before and during 4 weeks (a, n = 3), 2 weeks (b, n = 4; e, n = 3). and single (c, n = 4) pegIFNα treatment. Horizontal gray bars indicate pegIFNα treatment duration and dosage in µg/kg (**a**,**b**). Arrow indicates time point of the single 30 µg/kg pegIFNα injection (**c**). One day after last dosage mice were sacrificed and viral load was determined in liver and bile (**d**). Non-treated infected mice were added as control (**d**). X-axes indicates number of pegIFNα injections (**d**). HBV DNA was measured in serum of HBV gtA infected human-liver chimeric mice before and during pegIFNα treatment, and one day after last treatment mice were euthanized and intrahepatic HBV DNA was measured (**f**, n = 6). ^#^indicates 3 µg/kg pegIFNα dosages. All mice were transplanted with the same hepatocyte donor (HD2, **a**–**f**). Y-axes indicate log HEV RNA IU/gram (**a**–**c**,**d** left panel, e), log HEV RNA IU/ml (d right panel), and log HBV DNA copies/ml (**f**). X-axes indicate days post infection (**a**–**c**,**e**–**f**).

Baseline ISG expression in hepatocytes has been shown to predict the response to IFNα treatment in chronic HCV infected patients^30–32^. As HEV did not induce an ISG response *in vivo*, we examined the HEV-sensitivity to pegIFNα treatment. As a negative antiviral control, we applied the same treatment to HBV gtA infected mice, which has been shown to only slightly reduce serum HBV DNA levels in a similar humanized mouse model^33^. After 1 to 2 pegIFNα injections, HEV gt3 RNA became undetectable in feces of all treated animals (Fig. 3a–c). Complete viral clearance in liver and bile was observed in all mice at euthanasia 24 hours after 4 or 8 pegIFNα injections (Fig. 3d). To examine whether a single dose of 30 µg/kg pegIFNα would suffice to clear HEV gt3 *in vivo*, 4 animals received one injection after 6 weeks of ongoing HEV gt3 replication and were observed for an additional 4 weeks. This led to a complete viral clearance in 2 out of 4 mice and relapse in feces in the remainder 2 (Fig. 3c+d). Four weeks after the initial single pegIFNα dose, the latter 2 animals received repetitive 10-fold lower pegIFNα doses for 2 weeks. Again a steep decline in fecal HEV RNA loads was noted, but HEV RNA reemerged in feces and was detectable in bile and liver at euthanasia one day after the second pegIFNα treatment course (Fig. 3c+d). The high *in vivo* HEV IFNα sensitivity was corroborated in HEV gt1 infected animals. Again rapid suppression of HEV replication was noted in feces (Fig. 3e), liver and bile (data not shown) after a 2 week treatment course with 30 µg/kg pegIFNα. In contrast, a similar treatment regimen of HBV gtA infected mice induced a maximum decline of 0.7 ± 0.2 log HBV DNA copies/ml in serum with high intrahepatic viral loads at necropsy (6.9 ± 0.6 log HBV DNA copies/gr liver) (Fig. 3f). Non-treated HEV gt1, HEV gt3 and HBV infected mice never showed spontaneous viral clearance (Fig. 1b–d and Suppl. Fig. 2a–c), nor was loss of human chimerism in pegIFNα-treated animals observed, based on persistent detection of human albumin levels in mouse serum (data not shown). These data indicate that HEV, but not HBV is highly sensitive to pegIFNα in humanized mice.

### Upregulation of intrahepatic ISG and serum CXCL10 upon pegIFNα treatment

**Figure 4:**
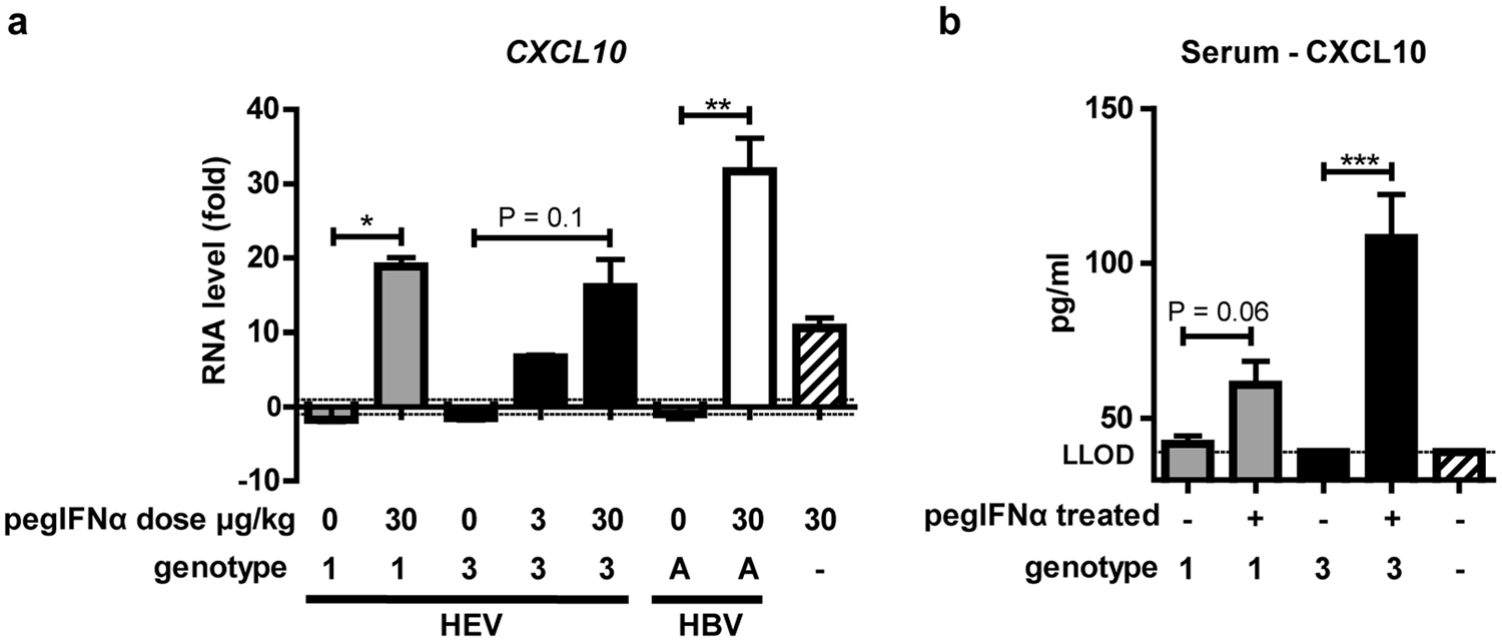
CXCL10 transcripts and protein are induced after pegIFNα treatment in HEV infected mice. RNA was isolated from non-treated and 2 weeks pegIFNα treated HEV gt1, gt3 and HBV infected mouse livers and was analyzed for the expression of human *CXCL10* (**a**). X-axes indicate treatment dosage and virus genotype (**a**). Given values on y-axes are RNA levels in fold changes over uninfected non-treated mice (**a**). Human CXCL10 levels were measured using ELISA in mouse serum of uninfected, HEV-infected and HEV-infected pegIFNα treated mice (**b**). Dotted line indicates lower limit of detection (LLOD) (**b**). Gray bars indicate HEV gt1, black bars HEV gt3 and striped bar uninfected (a + b).

To examine whether pegIFNα induced HEV clearance was associated with an induction of human hepatocyte ISG responses, human specific transcript levels of several innate immune response genes were studied in chimeric livers of HEV gt3, HEV gt1 and HBV gtA infected and pegIFNα treated animals. PegIFNα treatment led to an 20-fold increase of *CXCL10* transcription, conjointly with induction of *TLR3*, *DDX58, STAT1*, *CXCL9*, *ISG15*, *RSAD2*, *OAS1*, *MX1*, and *IFIT1* genes in the livers of HEV infected humanized mice (Fig. 4a and Suppl. Fig. 3). In addition, treatment was associated with an increase in serum human CXCL10 levels of HEV gt1 and gt3 infected mice (59 ± 10 and 108 ± 14 pg/ml, respectively, Fig. 4b). Interestingly, intrahepatic *CXCL10* expression levels remained elevated (3.4-fold compared to HEV-infected non-treated mice) 4 weeks after a single pegIFNα dose in the two mice that cleared HEV. Similar to previous reports, HBV persistence *in vivo* was not due to absence of an ISG response, as *IFIT1*, *ISG15*, *MX1*, *STAT1*, and *CXCL10* all were strongly induced (Fig. 4a and data not shown)^33^. Overall, HEV but not HBV was found to be sensitive to pegIFNα induced hepatocyte-specific innate responses *in vivo*.

## Discussion

Despite increasing reports on acute and chronic Hepatitis E Virus infections in Europe, antiviral treatment options are limited and immunological determinants of viral persistence remain largely unexplored^5^. Here we aimed to study baseline and therapeutically induced innate immune responses in a recently established humanized mouse model for chronic HEV infection. We demonstrate that (1) HEV is highly sensitive to pegIFNα treatment *in vivo*; (2) HEV infection in human hepatocytes doesn’t elicit an innate immune response; (3) HEV gt1 presents higher viral loads compared to HEV gt3.

HEV gt1 and gt3, but not HBV, showed to be highly sensitive to pegIFNα treatment in immune deficient human liver chimeric uPA^+/+^NOG mice. Viral clearance in feces, liver and bile was achieved after 4 and 2 weeks treatment and even after a single pegIFNα injection in 2/4 mice. PegIFNα associated viral clearance was accompanied by an increase of intrahepatic human ISGs and serum CXCL10 levels. In line with our data (Fig. 3f), the antiviral potency of pegIFNα against other hepatotropic viruses was less pronounced in similar humanized mouse models. PegIFNα reduced HBV viremia by 2.5 log IU/ml and HCV loads with 2.3 log IU/ml after 12 and 4 weeks of treatment respectively, without clearing the infection^33–35^. Successful IFNα treatment in immunocompetent woodchucks chronically infected with woodchuck hepatitis virus (a model for chronic HBV infection), is associated with an intrahepatic IFN-γ and NK/T cell gene signature, but not an ISG signature^36^. All together this suggests that pegIFNα has a strong direct anti-viral effect against HEV, whereas HBV and HCV require the immune system to achieve viral clearance or complete suppression.

Ongoing HEV gt1 or gt3 replication did not elicit human-innate immune responses in humanized livers of 30 uPA^+/+^NOG mice, irrespective of the infection duration, the human hepatocyte donor or viral isolate used. We specifically addressed the genomic response of human hepatocytes to HEV without that of infiltrating immune cells in our profound immune deficient uPA^+/+^NOG mice. In addition, we carefully eliminated cross hybridizing probes by including non-chimeric mice. After prolonged HEV gt3 infection for more than 3 months, significantly lower expression levels of *STAT1*, *RSAD2* and *MX1* compared to uninfected controls were observed, suggesting possible viral interference with the host’s cell innate immune signaling. Hepatotropic pathogens have developed different methods to evade innate immune defenses^37^. In our model, expression of *TLR3* and *DDX58* was detected in all HEV-infected chimeric livers indicating that these host sensing molecules were not counteracted at the transcription level (Fig. 2a). Several studies in HEK293T, A549 and Huh7 cells have suggested that HEV can directly interfere with phosphorylation of STAT1 and the induction of IFNα^20, 21, 38^. However, most of these studies use non-physiological HEV-infection models or are influenced by defects in the innate signaling of target cells^22^. Our findings indicate possible HEV mediated innate immune inhibitory effects in primary human hepatocytes. Further studies in differentiated human hepatocytes are required to determine how HEV is able to prevent immune sensing or disrupt innate signaling and how this influences viral fitness.

In contrast to our findings, one recent study infected a similar, but less profound immunodeficient uPA-SCID mouse model with the same HEV gt1 strain (Sar-55) and showed elevated ISG expression in 2 HEV-infected mice compared to one control animal^28^. While the impact of the hepatocyte donor type on expression levels cannot be disregarded as shown here (Suppl. Fig. 1), remnant mouse natural killer cell and Kupffer cell activity in the SCID compared to the NOG background might have contributed to the observed differences^39, 40^. The role of infiltrating innate immune cells in the liver during HEV-clearance was recently shown in the chimpanzee model^41^. In HEV-infected chimpanzees the intrahepatic expression levels of *BST2* (present in monocytes, macrophages and dendritic cells) and not those of the adaptive immune system, corresponded with the expression kinetics of several ISG’s, including *CXCL10*, *ISG15* and *OAS1* ^41, 42^.

An important finding of our study was that during both HEV gt1 and gt3 infections, no innate immune responses were induced despite higher HEV gt1 viral loads in mouse feces, bile and liver. These observations point to an intrinsic phenotypical difference of the distinct HEV genotypes, but cannot explain the different immune pathogenesis seen in patients, who have a strikingly different clinical presentation. Not only is disease severity higher in HEV gt1 infections, but also chronicity rates for HEV gt1 are found to be low or even zero. Possibly, different amounts of viral antigens or epitopes, could induce different magnitudes of natural killer cell or HEV-specific T cell responses resulting in respectively more clinical disease or less chronic infections for the different genotypes^43, 44^.

The clinical experience with pegIFN based therapies for chronic HEV is minimal. Eight cases have been published of which 5 showed a suppression of viremia at the first measured timepoint after initiation of pegIFNα treatment. PegIFNα treatment in HEV infected humanized mice modelled the viral decline seen in these 5 patients. It remains however unclear why some chronic HEV patients show slow viral declines upon IFN-treatment. We observed a viral relapse in feces, liver and bile of 2 humanized mice after a second pegIFNα treatment course (Fig. 3c+d). While animals received a 10-fold lower pegIFNα dose, the relapse might be partially ascribed to elevated intrahepatic ISG levels before retreatment. Increased *CXCL10* levels were measured in the liver of 2 mice 4 weeks after a single pegIFNα injection, which corresponds to the timepoint at which retreatment was given to the remainder mice of that group. Since in chronic HCV patients the virologic response to pegIFNα is associated with low baseline ISG expression levels^31^, it would be interesting to examine whether this holds true for chronic HEV patients as well.

In conclusion, despite higher viral loads for HEV gt1 in human-liver chimeric mice, both HEV gt1 and gt3 do not induce an intrahepatic innate immune response. HEV, but not HBV, is highly sensitive to pegIFNα treatment in humanized mice.

## Material and Methods

### Ethics, consent and permissions

The use of patient material was approved by the medical ethical committees of Erasmus Medical Center and Antwerp University Hospital. Informed consent was obtained from all subjects. All animal work was conducted according to relevant Dutch national guidelines. The study protocol was approved by the animal ethics committee of the Erasmus Medical Center (DEC nr. 141-12-11).

### Mouse origin and genotyping

Urokinase-type plasminogen activator (uPA)/NOD/Shi-*scid*/IL-2Rγ^null^ (NOG) mice were kindly provided by the Central Institute for Experimental Animals (Kawasaki, Japan)^45^. Mice were bred at the Central Animal Facility of the Erasmus Medical Center. Zygosity of mice was determined as described previously^15^. Mice were co-housed with a maximum of 4 mice per individually ventilated cage and were fed normal chow *ad libitum*.

### Human hepatocyte transplantation

Six to twelve week old male uPA-homozygous mice were transplanted as described previously^46^. In short, mice were anesthetized and transplanted via intrasplenic injection with 0.5 × 10^6^ to 2 × 10^6^ viable commercially available cryopreserved human hepatocytes from 1 of 3 donors (Corning, NY, USA; Lonza, Basel, Switzerland; Table 1). Graft take was determined by human albumin in mouse serum using an ELISA with human albumin cross-adsorbed antibody (Bethyl laboratories, Montgomery, TX, USA) as previously described^15^.

**Table 1:** Hepatocyte donors.

Donor ID	Gender	Age	Race
HD1	Male	2 years	Caucasian
HD2	Female	2 years	Caucasian
HD3	Female	7 months	Caucasian

### Viral strains, mouse infection and treatment

HEV gt3 was derived from feces of one of two chronic HEV patients (HEV0069 and HEV0122) as described previously^15^. HEV gt1 Sar-55 was derived from feces of a Rhesus macaque that had been originally inoculated with the human Sar-55 strain^47^. Eight weeks after transplantation human-liver chimeric mice were inoculated intravenously (i.v.) with 200 µl feces suspension containing HEV gt3 (8.8 log IU/ml or diluted to 6.8 log IU/ml), HEV gt1 (7.9 log IU/ml or diluted to 6.2 log IU/ml) or 200 µl patient serum containing HBV gtA (7.7 log IU/ml). After viral inoculation, mice were housed individually. Mice were treated with a single subcutaneous pegIFNα-2a (30 µg/kg unless stated otherwise, Pegasys, Roche, Basal, Switzerland) injection or every 3–4 days for 2 or 4 weeks. Overview of viral isolates are shown in Table 2. An overview of experimental groups is shown in Table 3 and Suppl. Figure 4.

**Table 2:** Viral isolates.

Virus	Genotype	Strain/Isolate*	Source	Inoculum
HEV	1	Sar-55	Rhesus macaque feces	feces suspension
HEV	3	HEV0069*	Chronic HEV patient feces	feces suspension
HEV	3	HEV0122*	Chronic HEV patient feces	feces suspension
HBV	A		Chronic HBV patient serum	serum

**Table 3:** Overview of experimental groups.

Treatment	Chimeric liver	Virus	n=	Hepatocyte donor
None	no	None	3	n/a*
	yes	None	8	HD1, HD2
	yes	HEV gt1	10	HD1
	yes	HEV gt3 (HEV0069)	16	HD1, HD2, HD3
	yes	HEV gt3 (HEV0122)	4	HD1
	yes	HBV gtA	5	HD3
pegIFNα-2a	no	None	2	n/a*
	yes	None	2	HD2
	yes	HEV gt1	3	HD2
	yes	HEV gt3 (HEV0069)	11	HD2
	yes	HBV gtA	6	HD2

*n/a, not applicable.

### HEV RNA and HBV DNA detection

The presence of HEV RNA in mouse serum, feces, bile and liver was determined by an ISO15189:2012-validated, internally controlled quantitative real-time RT-PCR, described previously^7, 15^. Cycle threshold (*Ct)* values above 38 were considered background, which corresponds to a lower limit of detection of 2.16 log_10_ HEV RNA units/ml in undiluted human serum. HEV RNAs detected in samples with *Ct* values below 38 are indicated with their calculated values. HBV viral load was measured in mouse serum and liver using a dual target approach, using primers and probes targeting preS-gen, as described before^48, 49^, and the X gene (HBV XJfwd12 5′-ggtctgtgccaagtgtttgst-3′, HBV XJprobe 5′-FAM-acgcaacccccactggctggg-BHQ1–3′, HBV XJrev12, 5′-tycgcagtatggatcgsc-3′).

### RNA isolation of whole liver, generation of cDNA and real-time qPCR

Whole liver RNA was isolated using RNeasy mini kit (Qiagen, Hilden, Germany) including DNAse treatment according to manufacturer’s protocol starting with homogenization of liver tissue in RLT buffer. cDNA was generated by using an iScript cDNA synthesis kit (Bio-Rad Laboratories, Hercules, CA, USA) according to the manufacturer’s protocol. Human specific gene expression was measured using Taqman primer/probe quantitative PCR, in TaqMan® Gene Expression Master Mix (Thermo Fisher Scientific, Waltham, MA, USA). Primer/probe combinations were purchased from Thermo Fisher Scientific; *CXCL10* (Hs01124251_g1), *CXCL9* (Hs00171065_m1), *DDX58* (Hs01061436_m1), *GAPDH* (Hs00266705_g1), *IFIT1* (Hs01911452_s1), *ISG15* (Hs01921425_s1), *IFNA1* (Hs00855471_g1), *IFNA4* (Hs01681284_sh), *IFNB1* (Hs01077958_s1), *MX1* (Hs00895608_m1), *OAS1* (Hs00973637_m1), *RSAD2* (Hs00369813_m1), *STAT1* (Hs01013996_m1), *TLR3* (Hs01551078_m1). Expression of target genes was normalized to the expression of *GAPDH* using the formula 2^−ΔCt^, ΔCt = Ct^target^−Ct^GADPH^. cDNA from non-chimeric mouse livers was used as control to test cross-reactivity of housekeeping and target genes. Due to the difference in hepatocyte donor baseline expression levels of examined genes (Suppl. Fig. 1), fold changes of transcripts were calculated to those of non-infected humanized livers from mice transplanted with the identical hepatocyte donor.

### Cytokine measurement

Human CXCL10 was measured in 1:5 diluted mouse serum samples using the Human CXCL10/IP10 Quantikine ELISA Kit (R&D Systems, Minneapolis, MN, USA) according to manufacturer’s protocol.

### Nanostring analyses

RNA was isolated from chimeric mouse livers as described above. The nCounter GX human Immunology V2 Kit (NanoString Technologies, Seattle, WA, USA) was used to measure the expression of 594 human genes in the RNA of these samples. Following hybridization, transcripts were quantitated using the nCounter Digital Analyzer. Samples were run at the Johns Hopkins Deep Sequencing & Microarray Core facility. To correct for background levels, the highest negative control value for each sample was subtracted from each count value of that sample, as described previously^50, 51^. Following background subtraction, any negative count values were considered as 0. The geometric mean of 5 housekeeping genes provided by the company panel was calculated and used to normalize expression values. RNA from non-chimeric mouse livers was used as control to test cross-reactivity of genes. Fifty cross-reactive genes were removed prior to analyses of the data set. Non-expressed genes were defined as < 100 relative RNA counts and below four times the standard deviation in all samples.

### Statistics

Differences between groups were calculated using two tailed Mann-Whitney test or Kruskal-Wallis one-way ANOVA with Dunn’s all column comparison post-test (GraphPad Prism version 5.01; GraphPad Software). Differences were considered significant when P < 0.05. Results are presented as the mean ± SEM. Principal component analyses was performed on log 2 transformed data set and heatmap of IFN signaling/response genes was generated using Multi-experiment viewer (MeV) software version 4.9.

## Electronic supplementary material

Supplemental data
