# Supplementary material for: Interferon-alpha treatment rapidly clears Hepatitis E virus infection in humanized mice
Source: Sci Rep. 2017 Aug 15;7:8267. doi: 10.1038/s41598-017-07434-y (PMC5557905; doi:10.1038/s41598-017-07434-y)
Supplement: Supplementary file 1 — Supplemental data [file 41598_2017_7434_MOESM1_ESM.pdf]

Interferon-alpha treatment rapidly clears Hepatitis E virus infection in humanized mice

Martijn D.B. van de Garde, Suzan D. Pas, Gertine W. van Oord, Lucio Gama, Youkyung Choi, Robert A. de Man, Andre Boonstra, Thomas Vanwolleghem

Table of contents

Supplementary Fig. 1……..……………………………………………………………. 2

Supplementary Fig. 2……..……………………………………………………………. 3

Supplementary Fig. 3……..……………………………………………………………. 4

Supplementary Fig. 4……..……………………………………………………………. 5

Supplementary Table. 1……..………………………………………………………. 6

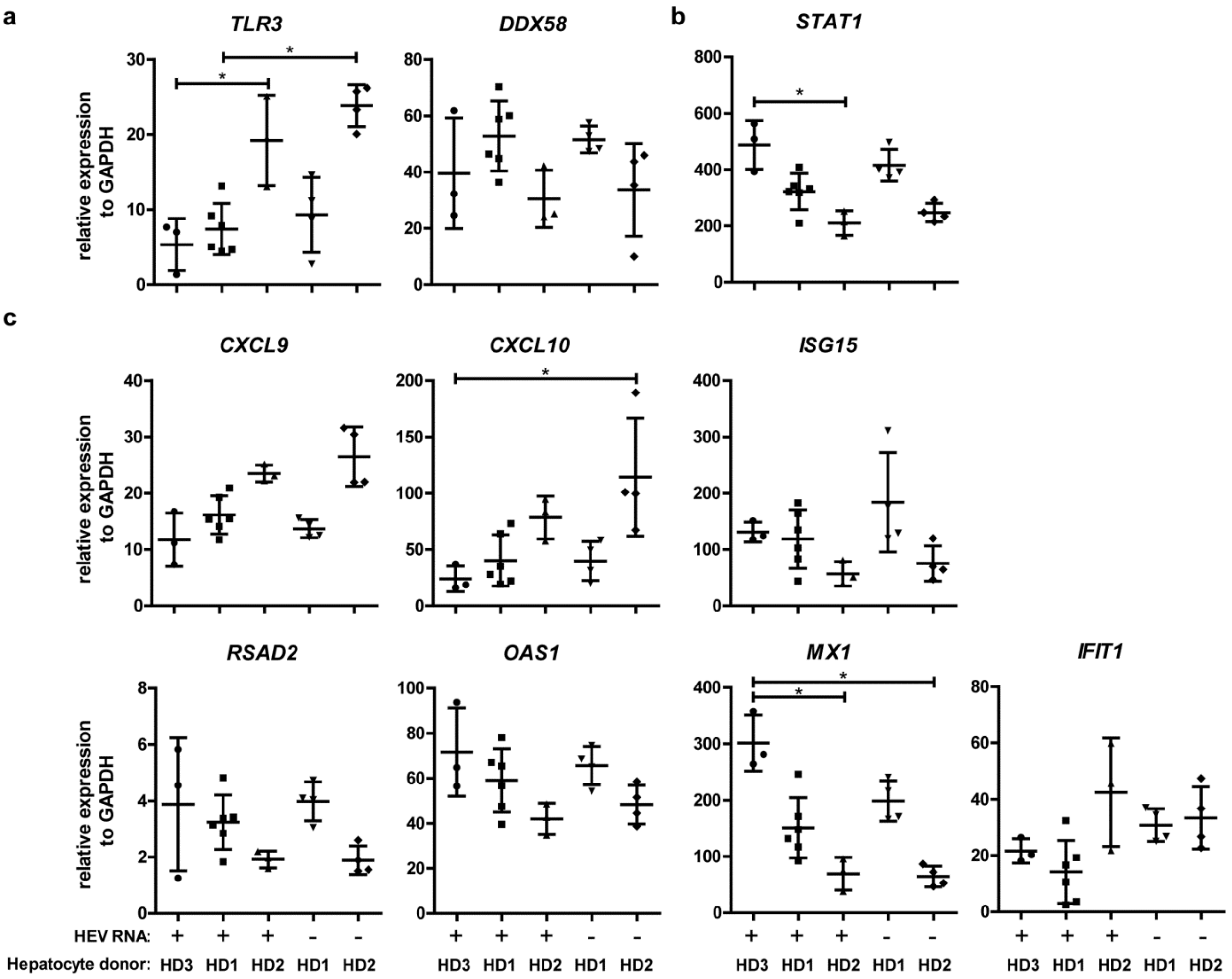

Supplemental figure 1. Hepatocytes donor is an important variable in the baseline gene expression profile. UPA$^{+/+}$NOG mice were transplanted with hepatocytes from one of three available donors (HD1, HD2, or HD3). Whole liver RNA was isolated from HEV infected and uninfected chimeric-mice (HEV RNA+ or – below X axis), and was analyzed for human specific gene expression of sensing molecules *TLR3* and *DDX58* (a), transcription factor *STAT1* (b), and interferon stimulated genes *CXCL9*, *CXCL10*, *ISG15*, *RSAD2*, *OAS1*, *MX1* and *IFIT1* (c) using qRT-PCR. Given values on y-axes are relative expression to *GAPDH*. X-axes shows HEV RNA presence and hepatocyte donor (a-c). Significance was assessed between all samples using Krukskal-Wallis one-way Anova with Dunnett's Multiple comparison test. * $P < 0.05$,

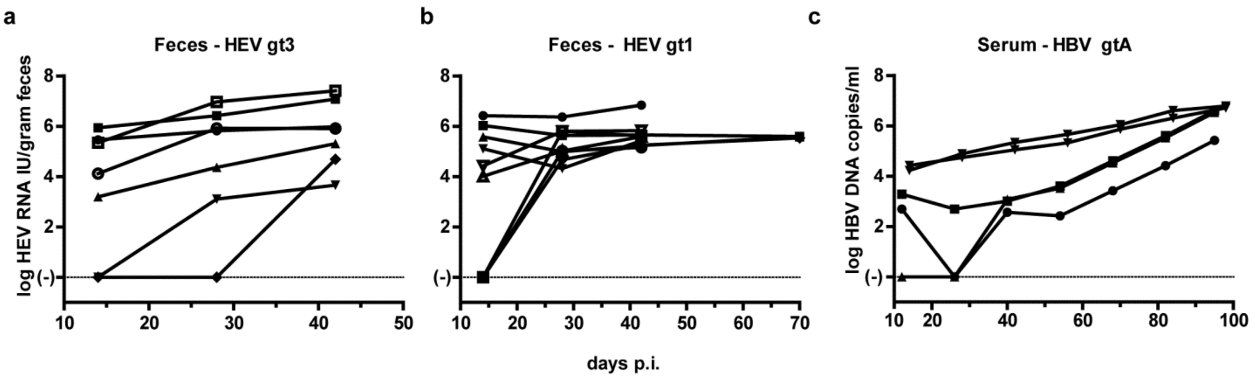

Supplemental figure 2. No drop in HEV RNA or viral clearance in gt1 or gt3 HEV, and HBV infected mice without pegIFNα treatment. HEV RNA was measured in feces of HEV gt3 (a, n=7) and HEV gt1 (b, n=9) inoculated mice. HBV DNA was measured in serum of mice challenged with HBV gtA (c, n=5). X-axes indicate days post infection until euthanasia (a-c). Y-axes indicated log HEV RNA IU/gram feces (a-b) or log HBV DNA copies/ml (c).

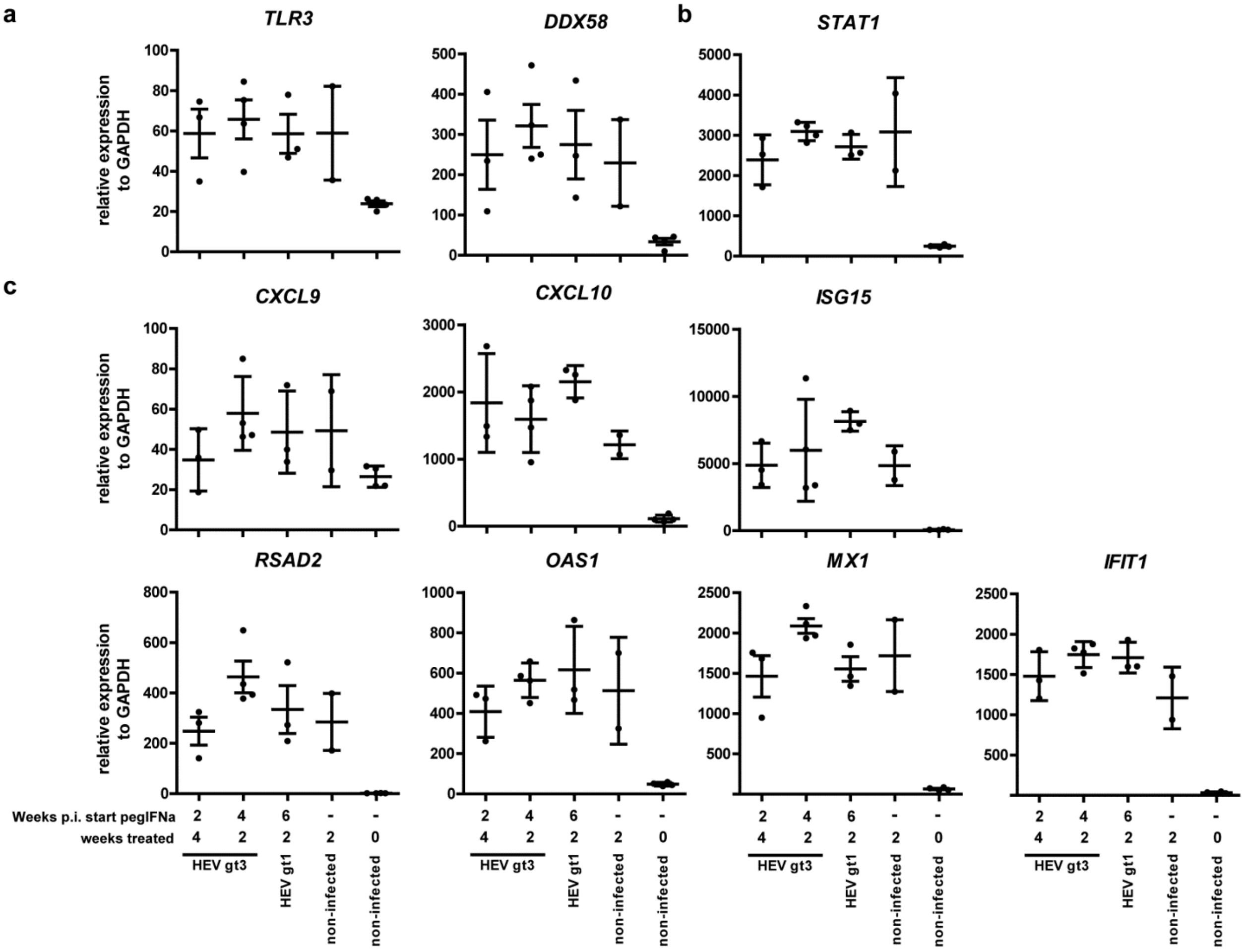

Supplemental figure 3. Genes related to interferon signaling and response are strongly upregulated after pegIFNα treatment in human-liver chimeric mice. Whole liver RNA was isolated from non-infected (n=4), non-infected treated (n=2), and HEV gt3 (n=7) and gt1 (n=4) infected pegIFNα treated chimeric mice, and was analyzed for human specific gene expression of sensing molecules *TLR3* and *DDX58* (a), transcription factor *STAT1* (b), and interferon stimulated genes *CXCL9*, *CXCL10*, *ISG15*, *RSAD2*, *OAS1*, *MX1* and *IFIT1* (c) using qRT-PCR. Given values on y-axes are relative expression to *GAPDH*. X-axes shows virus used for infection, time after infection at which treatment was started, and the duration of treatment in weeks (a-c).

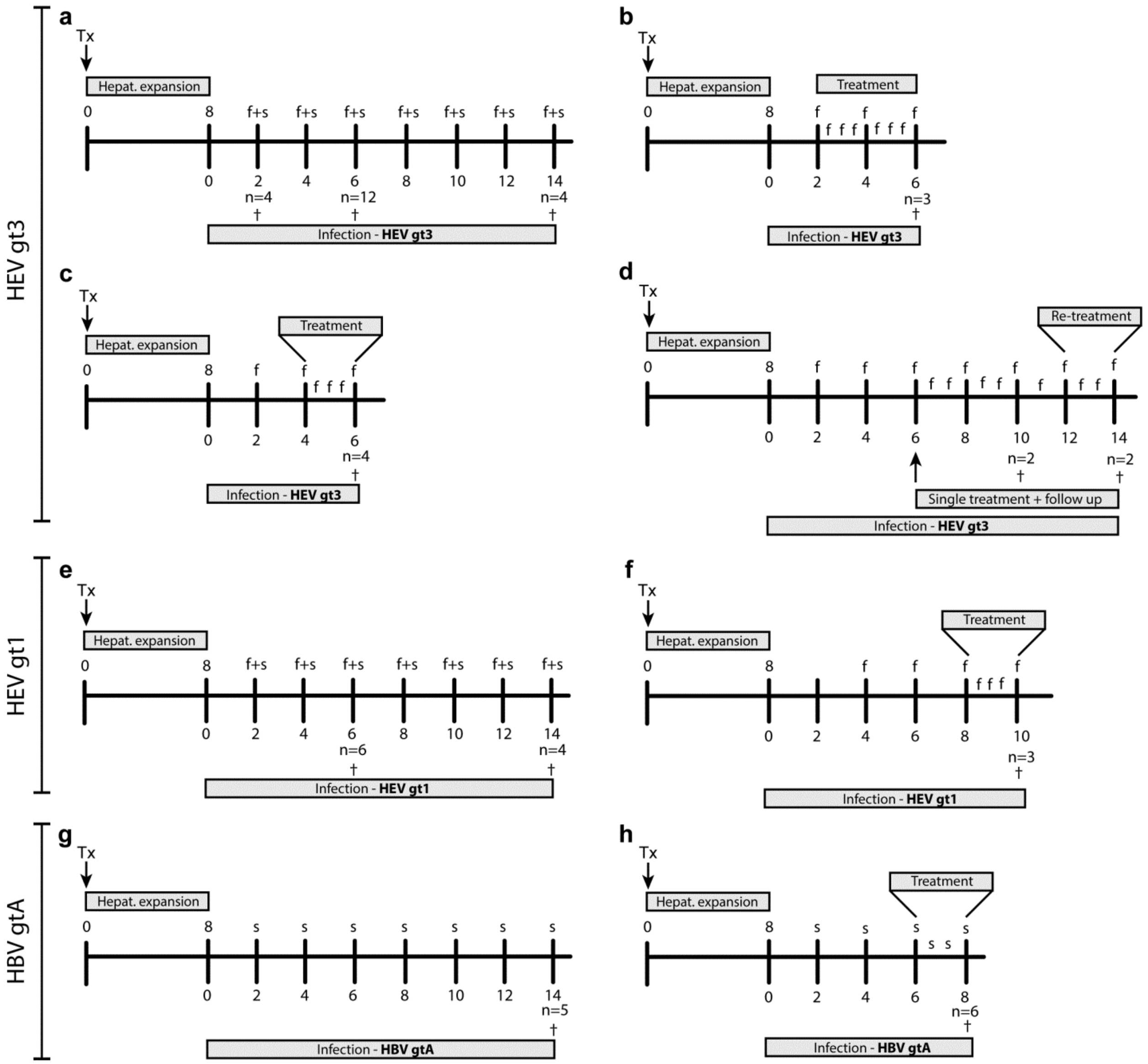

Supplemental figure 4. Overview of experimental groups of untreated and pegIFNα-treated infected mice. HEV gt3 infection experiment is illustrated and described in figure 1a-d, figure 2a-e and supplemental figures 1+2 (a). HEV gt3 treatment experiments are illustrated and described in figures 3a-d, figure 4a+b, and supplemental figure 3 (b-d). HEV gt1 infection experiment is illustrated and described in figure 1a-d, figure 2a, and supplemental figure 2 (e). HEV gt1 treatment experiment is illustrated and described in figure 3e, figure 4a+b, and supplemental figure 3 (f). HBV gtA infection experiment is illustrated and described in figure 4a, and supplemental figure 2 (g). HBV gtA treatment experiment is illustrated and described in figure 3f, and figure 4a (h). 'Tx' indicates human hepatocytes transplantation, 'f' indicates feces collection (a-f), 's' indicates serum collection (a-b, g-h). † indicates time of euthanization at which serum, feces, bile and liver were collected for HEV quantification, qPCR analysis,

Nanostring analysis or serum hAlbumin/cytokine quantification (a-h). Arrow below X-axes indicates single pegIFNα treatment dosage (d). Numbers above X-axes refer to weeks after human hepatocyte transplantation (transplantation time=0). Numbers below X-axes refer to weeks after HEV or HBV inoculation (inoculation time =0).

Supplemental Table 1. Relative RNA counts of HEV RNA negative and HEV RNA positive human-chimeric mouse livers

	HEV RNA negative				HEV RNA positive											
	-				HEV0069						HEV0122				HEV0069	
	Weeks post infection															
**Gene**	-	-	-	-	2	2	2	2	6	6	6	6	6	6	14	14
ABCB1	7185	7943	8097	8094	6281	7590	6825	5403	7486	5745	5900	6898	5692	7115	6135	6039
ABL1	658	657	825	613	781	692	664	1150	782	598	722	601	640	698	673	891
ADA	0	0	103	194	0	0	178	0	0	134	136	135	72	179	0	223
AHR	6062	5872	5465	6124	5340	5066	5068	4736	5243	5535	5052	6231	4457	5441	4993	3865
AICDA	0	0	0	0	0	0	0	0	0	0	0	0	0	0	0	0
AIRE	0	0	0	0	0	0	0	0	0	0	0	0	0	0	0	0
ARG1	33032	31145	33400	30453	30275	32797	30904	23796	28082	28455	28737	32468	27685	29498	26679	19362
ARG2	0	0	98	0	0	0	0	0	0	0	0	0	48	0	0	0
ARHGDIB	0	0	0	0	362	0	0	0	0	0	0	72	48	0	0	262
ATG10	631	732	751	760	1491	1349	858	1448	867	587	770	650	533	848	1162	1441
ATG12	172	0	123	183	0	0	219	0	0	141	157	181	139	162	296	262
ATG16L1	587	502	619	545	622	588	413	575	357	555	513	549	465	450	377	524
ATG5	1015	1091	1124	1163	1317	934	996	943	918	852	1199	1186	885	969	1050	1009
ATM	0	0	0	0	0	0	0	0	0	0	0	0	0	0	0	0
B2M	102415	103189	105469	97390	74285	87636	83424	59962	74704	82678	84505	103408	88345	77508	64160	48995
B3GAT1	0	0	0	0	0	0	0	0	0	0	0	0	0	0	0	0
BATF	0	0	83	0	420	467	178	0	0	76	147	79	43	0	234	393
BATF3	0	0	0	0	0	0	0	0	0	0	0	0	0	0	0	0
BCL10	1270	1327	1591	1446	1288	1677	1320	805	1589	1174	1246	1252	1197	1131	1315	1192
BCL2	344	620	486	445	1505	1037	1053	2851	816	645	660	450	314	767	1335	1677
BCL3	517	508	653	660	767	657	397	460	544	605	906	831	429	756	550	681
BCL6	772	1110	982	1105	434	1072	963	805	858	739	1115	1206	1048	1027	774	747
BID	0	186	157	199	0	0	202	0	229	105	0	187	82	214	275	262
BLNK	348	471	408	456	478	588	364	0	425	377	419	325	314	421	418	550
BST1	631	756	570	618	593	605	591	483	561	562	424	634	465	641	499	406
BST2	1990	1612	2033	1577	1230	1470	1668	1334	1631	1798	1785	1751	1775	1402	1060	865
BTK	0	0	0	0	0	0	0	0	0	62	0	0	48	0	0	223
BTLA	0	0	0	0	0	0	0	0	0	0	0	0	46	0	0	0
C1QA	0	0	0	0	0	0	0	0	0	0	0	0	0	0	0	0
C1QB	0	0	0	0	0	0	0	0	0	0	0	0	0	0	0	0
C2	9864	10225	10243	10871	9088	10788	10128	8070	10281	9979	9005	10200	10050	9435	11067	9026
C3	63736	56958	70252	67775	53112	63329	58601	48995	56487	61078	57715	64804	60657	56965	68257	49322
C4A/B	5115	3944	5009	5150	3907	4478	4987	3403	4580	6104	4633	5883	6390	5089	4148	2817
C4BPA	11786	10963	11146	10352	9725	9958	9529	8231	9041	11317	9806	11477	9681	10052	9977	8672
C5	3854	3162	3545	3699	2648	4011	3295	2575	3067	3483	3382	3824	3356	3197	3281	2555
C6	8643	7236	7940	7843	7829	7676	7562	7242	6483	8464	6911	8271	7556	6798	6634	6511
C7	0	0	0	0	0	0	0	0	0	0	0	0	0	0	0	0
C8B	9085	8340	8873	8586	7887	7382	7570	6001	7120	7590	6869	9145	7580	7242	6063	5633
C8G	5019	4526	5028	4945	4428	3942	4000	3058	4580	4089	4382	4819	4200	4812	3597	3013
C9	0	0	0	0	0	0	0	0	0	0	0	0	0	0	0	0
CAMP	0	0	0	0	0	0	0	0	0	0	0	0	0	0	0	0
CARD9	0	0	0	0	0	0	0	0	0	0	0	0	0	0	0	0
CASP1	526	583	638	602	449	674	445	690	408	381	571	535	360	358	326	380
CASP10	0	0	0	0	0	0	0	0	0	0	0	0	0	0	0	0
CASP2	437	415	457	382	579	622	461	0	544	511	414	434	353	427	611	563
CASP3	1301	1290	1552	1593	1042	1591	1198	1334	1402	1080	1099	1130	1031	1252	1029	825
CASP8	520	527	678	529	535	657	429	460	569	486	513	539	410	531	448	550
CCBP2	185	0	157	136	304	0	186	0	0	149	183	181	158	144	245	0
CCL11	0	0	0	0	0	0	0	0	0	0	0	0	0	0	0	223
CCL13	0	155	0	0	0	0	0	0	0	65	0	0	50	0	316	367
CCL15	2814	2617	2578	2902	2721	2178	2194	1862	2201	2302	2016	2434	2276	2020	2008	1795
CCL16	9485	8098	8986	9608	6816	8350	8453	6805	8930	9178	7895	8656	9823	8534	8142	7559
CCL18	0	0	0	0	0	0	0	0	0	0	0	0	0	0	0	0
CCL19	0	0	79	0	0	0	0	0	0	72	0	56	43	0	0	0
CCL2	0	0	123	136	0	0	0	0	0	105	0	168	96	0	0	275
CCL20	412	508	962	775	593	743	461	0	433	236	817	821	413	710	642	629
CCL22	0	0	0	0	0	0	0	0	0	0	0	0	43	0	0	0
CCL23	0	0	0	0	0	0	0	0	0	0	0	0	0	0	0	0
CCL24	0	0	0	0	0	0	0	0	0	0	0	0	0	0	0	0
CCL26	0	0	0	0	0	0	0	0	0	0	0	0	0	0	0	0
CCL3	0	0	0	0	0	0	0	0	0	0	0	0	0	0	0	0
CCL4	0	0	79	0	0	0	0	0	0	0	0	56	0	0	0	0
CCL5	0	0	0	0	0	0	0	0	0	0	0	0	0	0	0	0
CCL7	0	198	108	131	391	0	235	0	0	109	147	115	106	0	234	367
CCL8	111	254	231	204	434	0	219	529	246	210	209	154	151	283	285	511
CCND3	175	198	167	267	0	0	194	0	238	214	194	177	158	173	0	0
CCR1	0	0	83	136	391	0	0	0	0	80	0	69	65	0	275	0
CCR10	0	0	0	0	0	0	0	0	0	0	0	0	0	0	0	0
CCR2	0	0	0	0	0	0	0	0	0	0	0	0	0	0	0	0
CCR5	0	0	0	0	0	0	0	0	0	0	0	0	0	0	0	0
CCR6	0	0	108	0	0	0	0	0	0	58	0	59	0	0	0	0
CCR7	0	0	0	0	0	0	0	0	0	0	0	0	0	0	0	0
CCR8	0	0	0	0	0	0	0	0	0	0	0	0	0	0	0	0
CCRL1	0	0	0	0	0	0	0	0	0	0	0	0	0	0	0	0

CCRL2	0	0	0	0	0	0	0	0	0	0	0	0	0	0	0	0
CD14	3445	2970	3103	2997	2330	2783	3069	2161	2447	3197	3529	3380	4294	2724	3597	2345
CD160	0	0	0	0	0	0	0	0	0	0	0	0	0	0	0	0
CD163	0	0	83	0	0	0	0	0	0	0	0	0	0	0	0	0
CD164	5558	5810	5259	5836	4761	5463	4591	4069	4852	4647	5188	5328	4303	5014	5156	4664
CD19	0	0	0	0	0	0	0	0	0	62	0	0	0	0	204	262
CD1A	0	0	0	0	0	0	0	0	0	0	0	0	0	0	0	0
CD1D	428	335	368	440	0	501	389	483	501	424	366	342	329	387	306	275
CD2	0	0	0	0	0	0	0	0	0	0	0	0	0	0	0	0
CD209	0	0	0	0	0	0	0	0	0	0	0	0	0	0	0	0
CD22	0	0	0	0	0	0	0	0	0	0	0	0	0	0	0	0
CD244	0	0	0	0	0	0	0	0	0	0	0	0	0	0	0	0
CD247	0	0	0	0	0	0	0	0	0	62	0	0	0	0	0	0
CD27	0	0	0	0	0	0	0	0	0	0	0	0	0	0	0	0
CD274	0	0	83	0	0	0	0	529	0	0	0	0	48	156	0	0
CD276	2051	2108	2494	2357	1881	2317	2202	1701	2192	2001	2126	2145	1924	2031	2058	2135
CD28	0	0	83	0	0	0	0	0	0	62	0	0	0	0	234	0
CD34	0	0	0	0	0	0	0	0	0	0	0	0	0	0	0	0
CD3D	0	0	0	0	0	0	0	0	0	0	0	0	0	0	0	0
CD3E	0	0	0	0	0	0	0	0	0	0	0	0	0	0	0	0
CD3EAP	117	0	147	157	0	0	0	0	0	101	162	115	151	138	0	301
CD4	0	0	0	0	0	0	0	0	0	0	0	0	0	0	0	0
CD40	348	254	358	283	449	0	494	483	297	348	304	309	271	335	306	275
CD40LG	0	0	0	0	0	0	0	0	0	0	0	0	0	0	0	236
CD44	175	322	290	299	926	830	478	943	416	257	393	279	192	306	581	786
CD45R0	0	0	0	0	0	0	0	0	0	0	0	0	0	0	0	0
CD45RA	0	0	0	0	0	0	0	0	0	0	0	0	0	0	0	0
CD45RB	0	0	0	0	0	0	0	0	0	0	0	0	0	0	0	0
CD46	6530	6492	6153	6653	5398	6137	5441	4897	6279	5111	5518	5837	5210	5972	6175	5921
CD48	0	0	0	0	0	0	0	529	0	0	0	0	0	0	0	354
CD5	0	0	0	0	0	0	0	0	0	0	0	0	0	0	0	0
CD53	0	0	0	0	0	0	0	0	0	0	0	0	0	0	0	0
CD55	98	0	79	0	0	0	0	0	0	112	126	105	86	0	0	0
CD58	670	738	756	969	926	951	712	736	731	605	722	621	511	785	774	852
CD59	10596	10621	10346	10981	8784	9284	9246	7495	9466	9689	9843	10939	8462	9729	8815	9000
CD6	0	0	0	0	0	0	0	0	0	0	0	0	0	0	0	0
CD7	0	0	0	0	0	0	0	0	0	0	0	53	0	0	0	0
CD70	0	0	0	0	0	0	0	0	0	0	0	0	0	0	0	0
CD74	415	676	614	414	796	674	688	713	484	609	623	710	391	554	591	734
CD79A	0	0	0	0	0	0	0	0	0	0	0	0	0	0	0	0
CD79B	0	0	0	0	0	0	0	0	0	58	126	53	0	0	0	275
CD80	0	0	0	0	0	0	0	0	0	0	0	59	0	0	0	0
CD81	9968	9090	10120	10022	7757	10321	9635	6805	7928	9638	9597	10696	10924	8356	9528	7493
CD82	98	0	137	168	0	0	0	0	0	98	0	135	125	0	204	223
CD83	0	0	0	0	0	0	0	0	0	0	0	0	43	0	0	0
CD86	0	0	0	0	0	0	0	0	0	0	0	0	0	0	0	0
CD8A	0	0	0	0	0	0	0	0	0	0	0	79	62	0	0	0
CD8B	0	0	0	0	0	0	0	0	0	0	0	0	0	0	0	0
CD96	0	0	0	136	333	0	162	460	0	0	0	66	43	0	0	354
CD97	132	0	88	141	0	0	0	0	0	83	0	62	48	0	0	0
CD99	12770	12686	12777	13144	11505	12137	11950	10093	13000	12085	11408	12338	11341	11454	12606	9982
CDH5	0	0	0	0	0	0	0	0	0	0	0	0	0	0	0	0
CDKN1A	1575	1631	2318	1975	1447	1677	1182	1242	1326	903	1492	1442	1502	1402	876	1087
CEACAM1	388	446	481	466	738	674	356	713	578	323	408	414	307	531	428	445
CEACAM6	0	0	0	0	0	0	0	0	0	65	0	59	0	0	0	0
CEACAM8	0	0	0	0	0	0	0	0	0	0	0	0	0	0	0	0
CEBPB	5044	5208	6437	4993	3604	4547	3935	3150	4291	2918	5277	5492	5337	4109	3801	2568
CFB	24974	22855	24237	24691	19725	21231	20500	17405	20928	23424	21392	25497	25095	21552	22480	17882
CFD	0	0	0	0	0	0	0	0	0	0	0	0	0	0	0	0
CFH	30353	27152	30326	29390	25384	24740	23496	20485	22032	23308	23361	28625	24224	23393	20259	18615
CFI	9236	8470	9428	9409	8133	9682	8064	5886	6594	8787	7555	9191	7872	7478	7674	5974
CFP	0	0	0	0	0	0	0	0	0	0	0	0	0	0	0	0
CIITA	0	0	0	0	0	0	0	0	0	0	0	0	0	0	0	0
CISH	0	0	0	0	0	0	0	0	0	76	0	0	55	0	0	0
CLEC4A	0	0	0	0	0	0	0	0	0	83	0	56	46	0	0	0
CLEC4E	0	0	0	0	0	0	0	0	0	0	0	0	0	0	0	0
CLEC5A	0	0	0	0	0	0	0	0	0	0	0	0	0	0	0	0
CLEC6A	0	0	0	0	0	0	0	0	0	0	0	0	0	0	0	0
CLEC7A	0	0	0	0	0	0	0	0	0	0	0	0	0	0	0	0
CLU	0	0	113	120	0	0	219	0	0	138	0	76	86	0	0	0
CMKLR1	0	0	0	0	0	0	0	0	0	0	0	0	0	0	0	0
CR1	0	0	0	0	0	0	0	0	0	0	0	0	0	0	0	0
CR2	0	0	0	0	0	0	0	0	0	0	0	0	0	0	0	0
CRADD	754	738	683	802	868	571	712	0	765	765	654	706	679	658	622	734
CSF1	0	0	0	0	0	0	0	0	0	76	0	69	74	0	0	0
CSF1R	0	0	0	0	0	0	0	0	0	0	0	0	0	0	0	0
CSF2	0	0	0	0	0	0	0	0	0	0	0	0	0	0	0	0
CSF2RB	0	0	0	0	0	0	0	0	0	0	0	0	0	0	0	0
CSF3R	0	0	0	0	0	0	0	0	0	0	0	0	0	0	0	0
CTLA4_all	0	0	0	0	0	0	0	0	0	0	0	0	0	0	0	0
CTLA4-TM	0	0	0	0	0	0	0	0	0	0	0	0	0	0	0	0
CTSC	2381	2356	2234	2447	1606	1608	2040	1747	1878	1689	1628	1794	1907	2025	1661	1638

CTSG	0	0	0	0	0	0	0	0	0	0	0	0	0	0	0	0
CTSS	2208	1811	2234	2075	1418	2005	1781	1517	1538	1816	1665	2119	1432	1720	1070	1402
CUL9	304	285	324	320	304	0	300	0	0	290	293	332	221	289	214	262
CX3CL1	1212	1377	1733	1525	1201	1418	1279	1127	1606	1359	1466	1403	1161	1241	1753	1389
CX3CR1	0	0	0	0	0	0	0	0	0	0	0	0	0	0	0	0
CXCL1	120	341	241	241	521	605	308	943	0	145	298	227	166	323	673	917
CXCL10	1624	2331	2200	1703	1983	2161	1935	1219	2243	1613	2450	2648	1223	2083	1050	1362
CXCL11	0	0	0	0	0	0	0	0	0	58	0	59	0	0	0	0
CXCL13	0	0	0	0	0	0	0	0	0	0	0	0	0	0	0	0
CXCL2	141	0	162	120	0	0	243	0	229	123	0	148	360	0	0	0
CXCL9	123	155	88	0	0	0	162	0	0	76	173	118	72	0	0	0
CXCR1	0	0	0	0	0	0	0	0	0	0	0	0	0	0	0	0
CXCR2	0	0	0	0	0	0	0	0	0	0	0	62	43	0	0	288
CXCR3	0	0	0	0	304	0	0	0	0	0	0	0	0	133	234	0
CXCR4	0	0	0	0	0	0	0	0	0	0	0	0	0	0	0	0
CXCR6	0	0	0	0	0	0	0	0	0	0	0	0	0	0	0	0
CYBB	0	0	0	0	0	0	0	0	0	0	0	0	0	0	0	0
DEFB1	643	471	476	775	593	692	502	644	484	602	586	575	592	594	571	472
DEFB103A	0	0	0	0	0	0	0	0	0	0	0	0	0	0	0	0
DEFB103B	0	0	0	0	0	0	0	0	0	0	0	0	0	0	0	0
DEFB4A	0	0	0	0	0	0	0	0	0	0	0	0	0	0	0	0
DPP4	7357	7397	7699	7780	5919	6967	6704	6553	6993	6445	6591	7174	6311	5949	7704	6380
DUSP4	0	0	0	0	0	0	0	0	0	0	0	0	0	0	0	0
EBI3	0	0	0	0	0	0	0	0	0	0	0	0	0	0	0	0
EDNRB	0	0	108	0	405	0	186	483	0	76	136	62	0	156	0	223
EGR1	378	558	638	325	420	501	559	0	875	493	728	391	556	225	1070	629
EGR2	0	0	98	0	0	0	0	0	0	0	0	0	43	0	0	0
ENTPD1	0	0	0	0	0	0	0	0	0	0	0	0	0	0	0	0
EOMES	0	0	0	0	0	0	0	0	0	0	0	0	0	0	0	0
ETS1	0	0	216	194	0	0	0	0	0	120	147	138	77	138	0	0
FADD	172	167	187	152	0	0	170	0	0	159	141	141	158	225	0	0
FAS	0	0	133	0	0	0	0	0	0	0	126	62	46	0	0	0
FCAR	0	0	0	0	0	0	0	0	0	0	0	0	0	0	0	0
FCER1A	0	0	0	0	0	0	0	0	0	0	0	0	0	0	0	0
FCGR1A/B	0	0	0	0	0	0	0	0	0	0	0	0	0	0	0	275
FCGR2A	0	0	0	0	0	0	0	0	0	0	0	0	0	0	0	0
FCGR2A/C	0	0	0	0	0	0	0	0	0	65	0	105	0	0	0	0
FCGR2B	0	0	0	0	0	0	0	0	0	0	0	0	0	0	0	0
FCGR3A/B	0	0	113	0	0	0	0	0	0	0	0	53	0	0	0	0
FCGRT	8332	8495	8465	8330	7410	7711	8291	5472	7205	8964	7675	8886	8957	7784	8479	6511
FKBP5	993	905	1021	1137	941	1539	802	552	1130	569	880	1087	1027	1154	1060	747
FN1	100050	81642	107065	107930	80319	95952	93229	77504	100085	88261	88259	98613	84894	92649	89647	81824
FOXP3	0	0	0	0	0	0	0	0	0	0	0	0	0	0	0	0
FYN	0	0	0	0	0	0	0	0	0	0	0	0	0	0	0	0
GATA3	0	0	113	0	0	0	0	0	0	76	0	0	0	0	234	0
GBP1	572	670	1488	985	579	778	729	483	476	591	743	874	770	594	540	563
GBP5	0	0	0	0	0	0	0	0	0	62	0	0	46	0	0	0
GFI1	0	0	93	157	0	0	0	0	0	0	0	62	84	133	306	262
GNLY	0	0	0	0	0	0	0	0	0	0	0	0	0	0	0	0
GP1BB	0	0	0	0	0	0	0	0	0	0	0	0	43	0	0	0
GPI	4263	3931	4100	4563	2373	3752	3919	3012	3977	4034	4157	4517	4366	4322	3985	3812
GPR183	0	0	0	0	0	0	0	0	0	0	0	0	0	0	0	0
GZMA	0	0	0	0	0	0	0	0	0	0	0	0	0	0	0	0
GZMB	0	0	0	0	0	0	0	0	0	0	0	0	0	0	0	0
GZMK	0	0	0	0	0	0	0	0	0	0	0	0	0	0	0	0
HAMP	483	316	417	393	0	0	316	0	0	210	220	332	429	208	0	0
HAVCR2	0	0	0	0	0	0	0	0	0	0	0	0	0	0	0	0
HFE	178	0	231	194	0	0	0	0	0	170	168	135	130	196	204	0
HLA-A	10270	9741	10189	9875	7323	9077	8550	5794	8794	10189	10481	12128	11031	9008	9314	7074
HLA-B	4767	3739	4292	4097	2952	4754	3813	2529	2753	4473	4660	6113	4860	3185	3913	2306
HLA-C	3811	3373	3555	3384	2981	3544	3530	2138	3254	4256	3722	4316	4778	3762	3322	2817
HLA-DMA	108	161	142	136	318	0	0	0	0	112	0	89	74	0	234	236
HLA-DMB	0	0	0	0	0	0	0	0	0	0	0	66	53	0	0	0
HLA-DOB	0	0	0	0	0	0	0	0	0	62	0	0	0	0	214	223
HLA-DPA1	0	0	0	0	0	0	0	0	0	0	0	0	0	0	0	0
HLA-DPB1	0	0	0	0	0	0	0	0	0	0	0	0	0	0	0	0
HLA-DQA1	0	0	0	0	0	0	0	0	0	0	0	0	0	0	0	0
HLA-DQB1	0	0	0	0	0	0	0	0	0	0	0	0	0	0	0	0
HLA-DRA	0	0	0	0	0	0	0	0	0	0	0	0	0	0	0	0
HLA-DRB1	0	0	0	0	0	0	0	0	0	0	0	0	0	0	0	0
HLA-DRB3	0	0	0	0	0	0	0	0	0	0	0	0	0	0	0	0
ICAM1	126	192	196	241	333	0	186	0	0	76	230	207	132	225	306	328
ICAM2	0	0	0	0	0	0	0	0	0	0	0	0	0	0	0	236
ICAM3	1406	1482	1665	1697	2113	2023	1635	1471	1623	1377	1393	1458	1231	1489	1508	1585
ICAM4	0	0	0	0	0	0	0	0	0	0	0	0	0	0	0	0
ICAM5	0	0	0	0	0	0	0	0	0	0	0	0	0	0	0	0
ICOS	0	0	0	0	0	0	0	0	0	0	0	0	0	0	0	0
ICOSLG	514	539	741	650	420	605	453	0	484	308	639	542	600	652	530	393
IDO1	0	0	0	0	0	0	0	0	0	0	0	0	0	0	0	0
IFI16	0	0	0	0	0	0	0	0	0	0	0	0	43	0	0	236
IFI35	787	682	953	728	868	1037	810	0	756	721	675	729	657	641	622	655
IFIH1	551	496	486	498	637	571	389	506	493	522	361	503	465	398	346	314

IFIT2	729	502	466	529	550	657	413	552	442	671	346	463	410	398	0	314
IFITM1	0	0	93	0	0	0	0	0	0	58	0	59	77	0	0	236
IFNA1/13	0	0	0	0	0	0	0	0	0	0	0	0	0	0	0	0
IFNA2	0	0	0	0	0	0	0	0	0	0	0	0	0	0	0	0
IFNAR1	418	397	309	477	492	484	316	460	561	395	565	506	398	514	611	563
IFNAR2	1895	2164	2342	2190	2243	2351	1903	1747	2090	1823	1984	2001	1816	1904	2120	1533
IFNB1	0	0	0	0	0	0	0	0	0	0	0	0	0	0	0	0
IFNG	0	0	0	0	0	0	0	0	0	0	0	0	0	0	0	0
IFNGR1	2190	2400	2544	2541	1404	2075	1886	1563	1997	1541	2016	2161	1943	1852	1783	1546
IGF2R	1609	1414	1694	1750	1389	1850	1506	1242	1980	1428	1592	1856	1590	1760	1580	1742
IKBKB	304	360	349	377	420	0	453	598	229	236	393	384	252	392	316	511
IKBKE	0	0	118	0	0	0	0	0	0	0	0	72	67	0	0	0
IKZF1	0	0	0	0	0	0	0	0	0	0	0	0	0	0	0	0
IKZF2	95	316	177	178	535	0	397	782	340	268	162	168	82	335	530	550
IKZF3	0	0	0	0	0	0	0	0	0	0	0	0	0	0	0	0
IL10	0	0	0	0	0	0	0	0	0	0	0	0	0	0	0	0
IL10RA	0	0	0	0	0	0	0	0	0	0	0	0	0	0	255	328
IL11RA	221	180	182	136	0	0	202	0	0	250	225	269	209	208	275	236
IL12A	0	0	0	0	0	0	0	0	0	0	0	0	0	0	0	0
IL12B	0	0	0	0	0	0	0	0	0	0	0	0	0	0	0	0
IL12RB1	0	0	0	0	0	0	0	0	0	0	0	0	0	0	0	0
IL13	0	0	0	120	376	0	186	0	0	62	0	0	50	0	0	354
IL15	0	0	113	0	0	0	0	0	0	109	0	82	60	0	0	0
IL16	0	0	0	0	0	0	0	0	0	0	0	0	0	0	0	0
IL17A	0	0	0	0	0	0	0	0	0	0	0	0	0	0	0	0
IL17B	0	0	0	0	0	0	0	0	0	0	0	0	0	0	0	0
IL17F	0	0	0	0	0	0	0	0	0	0	0	0	0	0	0	0
IL18	0	0	79	157	0	0	0	0	0	65	157	115	70	156	265	236
IL18R1	335	415	363	388	420	536	421	621	510	366	319	292	295	294	479	367
IL18RAP	0	180	192	0	0	0	162	460	0	101	152	122	89	214	0	445
IL19	0	0	0	0	0	0	0	0	0	0	0	0	0	0	0	0
IL1A	0	0	0	0	0	0	0	0	0	0	0	0	0	0	0	0
IL1B	0	0	0	0	0	0	0	0	0	0	0	0	0	0	0	0
IL1R1	987	1023	1301	1378	941	1072	972	1035	1232	1037	1241	939	1125	825	1539	1140
IL1R2	412	403	388	388	420	743	453	0	391	468	440	381	410	364	560	354
IL1RL1	0	0	0	0	0	0	0	0	0	0	0	0	0	0	0	0
IL1RL2	141	167	152	126	0	0	0	0	0	94	147	154	67	0	224	0
IL1RN	215	161	231	267	0	0	0	0	0	174	215	200	302	162	224	0
IL2	0	0	0	0	0	0	0	0	0	0	0	0	0	0	0	0
IL20	0	0	0	0	0	0	0	0	0	0	0	0	0	0	0	0
IL21	0	0	0	0	0	0	0	0	0	0	0	0	0	0	0	0
IL21R	0	0	0	0	0	0	0	0	0	0	0	0	0	0	0	0
IL22	0	0	0	0	0	0	0	0	0	0	0	0	0	0	0	0
IL22RA2	0	0	0	0	0	0	0	0	0	0	0	0	0	0	0	0
IL23A	0	0	0	0	0	0	0	0	0	0	0	0	0	0	0	0
IL23R	0	0	113	131	0	0	0	0	0	58	0	0	43	0	0	0
IL26	0	0	0	0	0	0	0	0	0	0	0	0	0	0	0	0
IL27	0	0	0	0	0	0	0	0	0	69	0	56	48	0	0	0
IL28A	0	0	0	0	0	0	0	0	0	0	0	0	0	0	0	0
IL28A/B	0	0	0	0	0	0	0	0	0	0	0	0	50	0	0	0
IL29	0	0	0	0	0	0	0	0	0	0	0	0	0	0	0	0
IL2RA	0	0	0	0	0	0	0	0	0	0	0	0	0	0	0	0
IL2RB	0	0	108	0	333	0	162	0	0	69	0	62	58	0	0	314
IL2RG	0	0	0	0	0	0	0	0	0	0	0	0	0	0	0	0
IL3	0	0	0	0	0	0	0	0	0	0	0	0	0	0	0	0
IL4	0	0	0	0	0	0	0	0	0	0	0	0	0	0	0	0
IL4R	1110	1091	1341	1231	941	1694	1360	1012	1436	997	1126	1084	1147	1298	1233	917
IL5	0	0	0	0	0	0	0	0	0	0	0	0	0	0	0	0
IL6	0	0	0	0	0	0	0	0	0	0	0	0	0	0	0	0
IL6R	1353	1253	1650	1561	1259	1781	1457	1219	1266	1301	1382	1721	1631	1598	1141	943
IL6ST	8962	8978	9654	9650	7656	8973	7570	6920	8242	7286	8529	8961	7947	7934	8631	7441
IL7	0	0	0	0	0	0	0	0	0	62	0	62	48	0	0	262
IL7R	0	0	0	0	0	0	0	0	0	0	0	0	0	0	0	0
IL8	114	0	0	162	0	0	0	0	0	69	0	72	113	0	204	262
IL9	0	0	0	0	0	0	0	0	0	0	0	0	0	0	0	0
IRAK1	1642	1587	1522	1378	1143	1521	1336	1058	1410	1276	1539	1468	1221	1431	1335	1376
IRAK2	750	1141	1336	1435	984	1504	1085	1012	1105	681	1126	1068	1317	1235	1630	1074
IRAK3	0	0	103	0	0	0	0	0	0	0	0	0	0	0	0	0
IRAK4	314	248	368	320	347	484	291	0	255	301	377	299	259	346	357	354
IRF3	200	298	270	246	0	0	356	0	272	272	298	345	206	231	275	314
IRF4	0	0	0	0	0	0	0	0	0	0	0	0	0	0	0	0
IRF5	181	267	265	178	0	0	211	483	0	214	220	250	163	294	306	249
IRF7	551	595	496	424	376	0	421	552	484	526	571	414	561	508	387	288
IRGM	0	0	0	0	0	0	0	0	0	0	0	0	0	0	0	0
ITGA2B	0	0	0	0	0	0	0	0	0	0	0	0	0	0	0	0
ITGA4	0	0	0	0	0	0	0	0	0	0	0	0	0	0	0	0
ITGA6	1169	1153	1193	1477	1230	1400	1182	1311	1343	1069	1325	1399	1024	1310	1590	1362
ITGAE	301	391	358	288	376	501	372	0	314	236	267	351	189	312	326	328
ITGAL	203	205	187	189	0	0	251	0	0	236	204	220	182	167	245	223
ITGAM	0	0	0	0	0	0	0	0	0	0	0	0	0	0	0	0
ITGAX	0	0	0	0	0	0	0	0	0	0	0	0	0	0	0	0
ITGB1	7837	8402	8701	8859	6469	7296	7238	5794	8905	6952	7424	7437	6520	7813	7541	7153

ITGB2	95	229	79	0	0	0	0	0	0	87	0	76	122	0	0	223
ITLN1	0	0	0	0	0	0	0	0	0	0	0	0	0	0	0	0
ITLN2	0	0	0	0	0	0	0	0	0	0	0	0	0	0	0	0
JAK1	1793	1873	2072	2127	1795	2386	1627	1908	1818	1762	1880	2191	1854	2141	2140	1821
JAK2	200	217	192	215	0	0	227	0	0	188	215	187	206	179	234	288
JAK3	0	0	0	0	0	0	0	0	0	0	0	0	0	0	0	0
KCNJ2	0	0	113	141	449	0	0	460	0	199	141	72	53	162	296	393
KIR_Activating_Subgroup_1	0	0	103	126	0	0	0	0	0	76	0	59	62	150	204	262
KIR_Activating_Subgroup_2	0	0	0	0	0	0	0	0	0	0	0	0	0	0	0	0
KIR_Inhibiting_Subgroup_1	0	0	0	0	0	0	0	0	0	0	0	0	0	0	0	0
KIR_Inhibiting_Subgroup_2	0	0	0	0	0	0	0	0	0	0	0	0	0	0	0	0
KIR3DL1	0	0	0	0	0	0	0	0	0	0	0	0	0	0	0	0
KIR3DL2	0	0	0	0	0	0	0	0	0	58	0	59	0	0	0	0
KIR3DL3	0	0	0	0	0	0	0	0	0	0	0	0	0	0	0	0
KIT	218	316	221	314	810	1003	567	1747	552	228	319	283	115	490	815	629
KLRAP1	0	0	0	0	0	0	0	0	0	65	0	0	0	0	0	0
KLRB1	0	0	0	0	0	0	0	0	0	0	0	0	0	0	214	0
KLRC1	0	0	0	0	0	0	0	0	0	0	0	0	0	0	0	0
KLRC2	0	0	0	0	0	0	0	0	0	0	0	0	0	0	0	0
KLRC3	0	0	0	0	0	0	0	0	0	0	0	0	0	0	0	0
KLRC4	0	0	0	0	0	0	0	0	0	69	0	0	0	0	0	0
KLRD1	0	0	0	0	0	0	0	0	0	0	0	0	0	0	0	0
KLRF1	0	0	0	0	0	0	0	0	0	0	0	0	0	0	0	0
KLRF2	0	0	0	0	0	0	0	0	0	0	0	0	0	0	0	0
KLRG1	0	0	0	0	0	0	0	0	0	0	0	0	0	0	0	0
KLRG2	0	0	0	0	0	0	0	0	0	0	0	0	0	0	0	0
KLRK1	0	0	0	0	0	0	0	0	0	0	0	0	0	0	0	0
LAG3	138	161	118	173	0	0	194	0	0	145	162	108	91	167	296	0
LAIR1	0	0	0	0	0	0	0	0	0	0	0	0	0	0	0	223
LAMP3	0	0	98	0	0	0	0	0	0	62	0	76	0	0	0	0
LCK	0	0	0	0	0	0	0	0	0	0	0	0	0	0	0	0
LCP2	0	0	83	0	0	0	0	0	0	0	0	85	0	0	0	249
LEF1	101	149	83	0	0	0	0	0	0	72	0	0	86	144	0	249
LGALS3	1399	1649	1321	1855	1085	1055	931	1012	2124	1040	1366	1344	811	1818	1590	2004
LIF	329	570	516	534	970	778	567	851	561	420	455	332	281	508	927	799
LILRA1	0	0	0	0	0	0	0	0	0	0	0	0	0	0	0	0
LILRA2	0	0	0	0	0	0	0	0	0	0	0	0	0	0	0	0
LILRA3	0	0	0	0	0	0	0	0	0	0	0	0	0	0	0	0
LILRA4	0	0	0	0	0	0	0	0	0	0	131	0	0	0	0	236
LILRA5	0	0	0	0	0	0	0	0	0	0	0	53	55	0	0	328
LILRA6	308	632	506	576	1389	1158	664	1586	680	439	633	394	331	791	1233	1533
LILRB1	0	0	0	0	0	0	0	0	0	0	0	0	0	0	0	0
LILRB2	0	0	0	0	0	0	0	0	0	58	0	0	0	0	0	0
LILRB3	0	0	0	0	0	0	0	0	0	0	0	0	0	0	0	0
LILRB4	0	0	0	0	0	0	0	0	0	0	0	0	0	0	0	0
LILRB5	0	0	0	0	0	0	0	0	0	0	0	0	0	0	0	0
LITAF	3122	3007	3860	3232	2562	2870	2915	2138	3203	2675	3466	3219	3233	2810	3078	2725
LTA	0	0	0	0	0	0	0	0	0	0	0	53	0	0	204	0
LTB4R	0	0	103	0	0	0	170	0	0	87	0	66	50	150	0	0
LTB4R2	0	0	0	0	0	0	0	0	0	0	0	0	62	0	0	0
LTBR	2980	3069	3290	3437	2663	2714	2623	2276	2821	2599	2701	2970	2826	2914	3139	2397
LTF	0	0	0	0	0	0	0	0	0	0	0	0	0	0	0	0
LY96	0	0	0	0	0	0	0	0	0	0	0	0	0	0	0	0
MAF	191	149	118	141	0	0	275	0	0	127	220	164	101	225	224	262
MALT1	1224	1532	1601	1551	2287	1988	1660	3196	1487	1160	1550	1288	1173	1748	2487	2607
MAP4K1	0	0	0	0	0	0	170	0	0	0	0	0	48	0	0	0
MAP4K2	203	279	187	204	0	0	308	0	280	228	272	207	218	271	275	0
MAPK11	0	155	83	0	333	0	0	0	0	80	0	0	62	185	0	301
MARCO	0	0	0	0	0	0	0	0	0	0	0	0	0	0	0	0
MASP1	1024	1017	967	927	868	1106	850	805	756	761	733	867	633	791	805	799
MASP2	3678	2852	3275	3452	2344	2593	3247	2828	2914	3944	3057	3804	3821	3520	3292	2699
MBL2	2368	2226	2352	2268	1997	1798	1773	1517	1640	1704	1932	2076	1749	1777	1906	1271
MBP	532	1060	815	639	1606	1470	1117	2299	892	975	796	650	612	917	1223	1166
MCL1	7366	8253	12335	11426	7091	8731	7505	5334	7214	4687	9052	7963	7911	8459	6838	5057
MME	520	459	643	681	709	847	632	598	408	598	450	430	657	502	591	419
MR1	151	186	167	152	0	0	0	0	0	116	0	92	70	173	0	0
MRC1	0	0	0	0	0	0	0	0	0	0	0	0	0	0	0	0
MS4A1	0	0	0	0	0	0	0	0	0	0	0	0	0	0	0	0
MSR1	0	0	0	0	0	0	0	0	0	0	0	0	0	0	0	0
MUC1	0	0	0	0	0	0	0	0	0	0	0	0	0	0	0	0
MX1	8080	6163	7945	5501	3155	5429	3967	2069	6424	5154	4304	4306	5673	3745	1335	904
MYD88	1944	2077	1758	1818	1679	2040	1538	1448	1776	1751	1644	1573	1813	1489	1783	1611
NCAM1	0	211	206	236	564	519	170	713	365	91	147	102	101	202	316	445
NCF4	0	0	0	0	0	0	0	0	0	0	0	0	0	0	0	0
NCR1	0	0	0	0	0	0	0	0	0	0	0	0	0	0	0	0
NFATC1	240	298	250	236	666	519	397	690	399	272	330	256	178	340	530	694
NFATC2	394	422	408	529	405	501	510	0	450	399	440	450	331	519	734	459
NFIL3	1436	1786	2382	1645	1476	1297	1255	1035	714	667	1576	1590	1142	600	744	1061
NFKB1	489	608	638	655	579	795	607	529	450	460	581	565	458	473	520	380

NFKB2	754	831	992	849	984	985	753	759	807	721	801	913	655	1027	846	721
NFKBIA	3586	3956	4257	3562	3575	4201	3902	3334	3220	2853	4523	4080	4025	4028	5146	2790
NFKBIZ	317	360	614	545	796	795	486	736	578	366	571	411	319	612	591	707
NLRP3	0	0	0	0	0	0	0	0	0	0	0	0	0	0	0	0
NOD1	0	0	0	0	0	0	0	0	0	0	0	66	0	0	0	0
NOD2	0	0	0	126	0	0	0	0	0	94	0	0	50	0	204	0
NOS2	0	0	0	0	0	0	0	0	0	0	0	0	0	0	0	0
NOTCH1	141	0	162	178	0	0	0	0	0	156	126	145	108	0	0	0
NT5E	1033	1166	913	1105	1274	1331	931	920	1266	743	900	782	595	1085	1090	1192
PAX5	0	0	0	0	0	0	0	0	0	0	0	0	0	0	0	0
PDCD1	0	0	0	0	0	0	0	0	0	0	0	0	0	0	0	0
PDCD1LG2	0	0	0	0	0	0	0	0	0	0	0	0	0	0	0	0
PDCD2	301	267	290	346	333	501	356	0	408	373	346	348	290	323	408	301
PDGFB	0	0	0	0	0	0	0	0	0	0	0	0	0	0	0	0
PDGFRB	154	217	196	199	0	0	162	0	229	156	257	243	151	208	560	367
PECAM1	0	0	88	136	0	0	0	0	0	0	0	0	79	0	0	0
PIGR	4506	4675	5117	4516	3386	3734	3902	2621	4597	3400	3785	4040	2989	4501	3597	3616
PLA2G2A	0	0	0	0	0	0	0	0	0	0	0	0	0	0	0	0
PLA2G2E	0	0	79	0	0	0	0	0	0	0	0	0	0	0	0	0
PLAU	0	0	0	0	0	0	0	0	0	0	0	0	0	0	0	0
PLAUR	0	0	0	0	0	0	0	0	0	0	0	0	0	0	0	0
PML	341	384	417	351	478	0	340	598	459	384	398	371	341	329	316	380
POU2F2	289	626	545	519	4255	3440	1020	2276	1113	892	932	552	213	710	2497	3210
PPARG	335	564	516	508	709	692	413	828	493	330	492	401	343	514	876	668
PPBP	0	0	0	0	0	0	0	0	0	0	0	0	0	0	0	0
PRDM1	0	0	0	0	0	0	0	0	0	0	0	56	46	0	0	0
PRF1	0	0	0	0	0	0	0	0	0	0	0	0	0	0	0	0
PRKCD	351	341	378	346	0	0	356	0	357	286	330	355	326	427	306	432
PSMB10	452	484	530	461	507	588	494	0	510	584	455	493	420	537	520	314
PSMB5	5911	6269	6295	6114	4819	5394	4680	4253	5446	4807	5173	5404	4294	5234	5391	4258
PSMB9	741	849	766	812	1042	1193	721	920	705	837	759	749	619	739	683	799
PSMD7	4770	4911	5067	4945	4067	4962	4000	3656	4384	4198	4251	4185	4015	4132	3740	3026
PTAFR	0	0	0	0	0	0	0	0	0	0	0	0	0	0	0	0
PTGER4	123	298	201	262	622	795	421	1311	357	174	356	161	137	265	469	668
PTGS2	0	0	0	0	0	0	0	0	0	0	0	0	0	0	0	0
PTPN22	0	0	0	0	0	0	0	0	0	0	0	0	0	0	0	0
PTPN6	348	409	329	382	449	0	340	0	391	326	246	319	314	346	397	341
PTPRC_all	0	0	108	0	0	0	243	506	0	0	0	0	67	0	296	0
PYCARD	92	0	118	173	0	0	170	0	0	134	0	102	74	0	0	0
RAF1	2842	3119	2902	3091	2619	2766	2882	2437	2821	2461	2843	2677	2300	2568	2415	2070
RAG1	0	0	98	120	0	0	0	0	0	58	0	59	0	0	0	0
RAG2	0	0	0	0	0	0	0	0	0	0	0	0	0	0	0	0
RARRES3	1064	1060	1277	980	1346	1020	1101	736	977	1185	843	1140	876	1125	907	799
RELA	858	738	913	938	651	743	745	897	867	696	969	838	926	894	866	511
RELB	274	248	334	356	362	0	502	0	348	163	262	253	166	346	296	367
RORC	471	453	584	623	651	864	470	874	442	580	529	578	722	473	805	694
RUNX1	0	0	147	189	0	0	0	0	0	65	0	92	103	0	0	0
S100A8	0	0	0	0	0	0	0	0	0	76	0	53	43	0	0	249
S100A9	0	0	0	0	0	0	0	0	0	0	0	0	48	0	0	0
S1PR1	231	217	241	210	362	467	291	0	0	178	194	191	218	260	336	380
sCTLA4	98	0	128	0	0	0	0	0	0	134	126	79	82	0	0	0
SELE	0	0	0	0	0	0	0	0	0	0	0	0	0	0	0	0
SELL	0	0	0	0	0	0	0	0	0	0	0	0	0	0	0	0
SELPLG	0	0	0	0	0	0	0	0	0	0	0	0	0	0	0	0
SERPING1	23645	19060	20751	21411	17352	20141	19156	15036	16382	23152	19371	25100	24867	18552	19444	15209
SH2D1A	0	0	0	0	0	0	0	0	0	0	0	0	0	0	0	223
SIGIRR	163	0	142	194	0	0	0	0	0	138	157	158	113	231	0	0
SLAMF1	0	0	0	0	0	0	0	0	0	0	0	0	0	0	0	0
SLAMF6	0	0	0	0	0	0	0	0	0	0	0	0	0	0	0	0
SLAMF7	0	0	0	136	0	0	162	0	0	80	0	59	0	0	285	249
SLC2A1	0	0	0	0	0	0	0	0	0	0	0	0	0	0	0	0
SMAD3	1384	1290	1247	1425	1259	1642	1158	1425	1546	1135	1618	1330	1046	1321	1416	1310
SMAD5	1799	2319	2504	2274	2576	2507	2065	2391	2736	1530	2366	2023	1454	2239	2089	1742
SOCS1	0	0	103	0	0	467	0	0	0	0	0	72	58	173	0	249
SOCS3	0	0	0	0	0	0	0	0	0	0	0	0	0	0	0	0
SPP1	843	620	805	1566	810	847	834	506	1444	627	801	798	547	929	540	799
SRC	464	558	437	477	651	519	397	0	552	417	408	420	271	346	459	367
STAT1	4109	3888	4144	4029	2012	3769	2850	1816	3713	3701	3262	3387	3022	3306	1641	1913
STAT2	1845	1662	1763	1755	1795	1677	1733	1448	1461	1620	1524	2089	1420	1743	1243	1074
STAT3	2427	2387	2789	2693	2533	2455	2388	2069	2541	2266	2309	2648	2262	2095	1865	1978
STAT4	231	434	280	346	478	501	348	529	408	265	356	312	197	300	510	668
STAT5A	0	0	103	0	0	0	0	0	0	76	0	85	48	0	0	0
STAT5B	861	769	884	775	564	951	680	598	816	939	743	828	679	866	866	917
TAGAP	0	0	0	0	0	0	0	0	0	0	0	0	0	0	0	0
TAL1	0	0	0	0	0	0	0	0	0	0	0	0	0	0	0	0
TAP1	369	422	447	382	579	605	421	0	416	304	372	328	302	352	397	419
TAP2	332	248	398	341	0	0	186	0	314	315	408	286	348	352	326	367
TAPBP	2645	2759	3005	2750	2388	2559	2842	2138	2999	2932	2942	3219	3054	3006	3108	2581
TBK1	1261	1333	1409	1336	883	1227	1077	1219	1045	942	885	1091	950	1102	1162	865
TBX21	0	0	0	0	0	0	0	0	0	0	0	0	0	0	0	0
TCF4	0	0	0	0	0	0	0	0	0	0	0	0	0	0	0	0
TCF7	357	304	363	356	0	0	356	575	255	355	419	325	393	306	459	367

TGFB1	0	161	137	141	0	0	194	0	0	94	152	105	89	156	316	354
TGFBI	9581	8799	8785	10787	6932	8420	7392	6415	9168	7902	8942	9132	10434	8050	8142	7048
TGFBR1	652	849	722	712	825	761	632	690	824	721	759	756	576	837	836	681
THY1	0	0	0	0	0	0	0	0	0	0	0	0	0	0	0	0
TICAM1	631	595	830	718	709	692	672	483	612	544	785	673	712	646	550	773
TIGIT	0	0	0	0	0	0	0	0	0	0	0	0	0	0	0	0
TIRAP	157	0	152	0	0	0	202	0	0	181	152	158	139	173	245	249
TLR1	289	248	280	393	492	0	340	0	306	391	215	296	216	329	387	380
TLR2	231	223	250	372	492	553	308	621	340	196	277	217	180	300	479	328
TLR3	547	515	653	508	492	0	356	575	518	526	482	555	365	410	326	511
TLR4	255	167	329	272	0	0	332	0	0	232	257	256	254	208	296	223
TLR5	0	0	0	0	0	0	0	0	0	0	0	0	0	0	0	0
TLR7	0	0	0	0	0	0	0	0	0	0	0	0	0	0	0	0
TLR8	0	0	0	0	0	0	0	0	0	0	0	0	0	138	245	367
TLR9	0	0	79	0	0	0	186	0	0	0	0	53	60	133	296	301
TMEM173	0	0	0	0	0	0	0	0	0	0	0	0	0	0	0	0
TNF	0	0	0	0	0	0	0	0	0	0	0	0	0	0	0	0
TNFRSF10C	188	180	221	147	304	0	211	0	238	87	126	138	84	173	214	0
TNFRSF11A	0	0	0	0	0	0	0	0	0	0	0	0	0	0	0	0
TNFRSF13B	0	0	0	0	0	0	0	0	0	0	0	0	0	0	0	0
TNFRSF13C	0	0	0	0	0	0	0	0	0	0	0	0	0	0	0	0
TNFRSF14	710	608	727	671	579	864	680	644	637	526	602	667	487	698	560	511
TNFRSF17	0	0	0	0	0	0	0	0	0	62	0	56	0	0	0	0
TNFRSF1B	203	0	241	131	0	0	194	0	0	91	162	145	182	0	0	0
TNFRSF4	0	0	0	0	0	0	0	0	0	0	0	0	0	0	0	0
TNFRSF8	0	0	0	0	0	0	0	0	0	0	0	0	0	0	0	0
TNFRSF9	0	0	0	0	0	0	0	0	0	0	0	0	0	0	0	0
TNFSF10	4075	4297	3693	3992	3025	3440	3927	3426	3654	6115	3838	4471	4126	3953	4066	3118
TNFSF11	246	236	339	330	376	0	267	0	357	156	220	204	158	317	265	301
TNFSF12	120	149	152	220	0	0	0	0	0	149	141	200	144	254	214	262
TNFSF13B	0	0	0	0	0	0	0	0	0	0	0	0	43	0	0	0
TNFSF15	0	0	0	0	0	0	170	0	0	0	0	0	0	0	0	223
TNFSF4	108	149	137	189	347	467	194	621	0	98	0	79	96	138	245	275
TNFSF8	0	0	83	0	0	0	0	0	0	0	0	0	0	0	0	0
TOLLIP	3122	3478	3334	3353	2735	3319	3085	2322	3033	3005	2801	3456	2994	3018	3424	3249
TP53	1329	1395	1385	1226	1925	1729	1279	2069	1444	1055	1272	1163	830	1246	1467	1428
TRAF1	0	0	0	0	0	0	0	0	0	83	0	76	0	0	0	0
TRAF2	317	310	491	361	478	484	348	460	365	279	335	388	247	433	448	354
TRAF5	0	0	0	0	0	0	0	0	0	0	0	0	0	0	0	0
TRAF6	458	670	604	650	637	813	583	667	612	431	497	568	415	589	611	1022
TYK2	1018	806	1021	875	897	1227	972	1012	816	841	880	1028	741	958	1080	852
UBE2L3	824	787	658	639	781	657	648	621	569	594	712	726	571	692	601	485
VCAM1	0	0	118	0	0	467	0	0	0	109	0	0	55	0	0	328
VTN	58400	57392	61242	58895	54125	57779	53403	43546	57779	69919	61187	67715	67561	64865	69500	62632
XBP1	7182	7366	7061	6920	6889	7382	6275	5610	6084	6180	6461	7145	6604	6532	5931	5987
XCL1	0	0	0	0	0	0	0	0	0	0	0	0	0	0	0	0
XCR1	0	0	0	0	0	0	0	0	0	0	0	0	0	0	0	0
ZAP70	0	0	0	0	0	0	0	0	0	0	0	0	0	0	0	0
